# Pilot of a novel patient-led intervention for postdischarge from hospital management of older patients’ care in general practice

**DOI:** 10.1136/fmch-2026-003981

**Published:** 2026-07-08

**Authors:** Rachel Spencer, Naomi Klepacz, Annabelle Louisa Long, Simon Edward Frank Spencer, Hema Mistry, Frances Griffiths, Jeremy Dale

**Affiliations:** 1 University of Warwick Medical School, Coventry, UK; 2 Health & Community Science, University of Exeter, Exeter, UK; 3 Statistics, University of Warwick, Coventry, UK

**Keywords:** General Practice, Community-Based Participatory Research, Patient-Centred Care, Health Literacy, Transitional Care

## Abstract

**Objective:**

General Practice Management After Transition Events (GP-MATE) is a codesigned patient-held tool to structure a dedicated postdischarge appointment for older patients. This pilot in routine general practice evaluated its feasibility (recruitment, tool use), acceptability (staff/patient views), potential outcomes (error, harm and readmission) and healthcare cost implications.

**Design and setting:**

Seven diverse general practices in the West Midlands, England, participated in this 4-year mixed-methods National Institute for Health and Care Research-funded study. A 20-minute GP-MATE appointment was offered to all patients aged >65 recently discharged from the hospital, supported by a paper-based communication tool sent to those who accepted. Three months of free-text electronic health records were reviewed for care use, intervention coverage, error, harm and readmission. Attributable healthcare costs were assessed. Patient/carer surveys and interviews with patients, carers and healthcare professionals explored experiences of delivery and use.

**Participants:**

40 patients (mean age 75 years; 53% female) attended GP-MATE appointments, and 29 returned surveys. 17 patient-held GP-MATE forms were retrieved and analysed. Seven patients/carers and nine healthcare professionals were interviewed.

**Results:**

Care appeared safe, with tentative signs of being two-thirds less error prone (*X*^2^=3.48 on 1df, p=0.06); no harms were detected. Postdischarge general practice use was high, with 115 admission-related contacts; almost two-thirds of the cohort had already received discharge-related care before GP-MATE. Despite this, GP-MATE appointments addressed persistent gaps in discharge information, leading to meaningful changes in care (mean=2.6 per patient), including new referrals and medication changes. Intervention costs represented just over half of the total mean attributable general practice care costs. Staff and patient/carer experiences indicate acceptability, though some unwell or confused patients struggled to differentiate the GP-MATE intervention from usual care. The perceived benefits were lower for elective or less complex admissions. Practices reported mixed experiences, including administrative difficulties in booking appointments and high non-attendance rates.

**Conclusion:**

GP-MATE was feasible to deliver, acceptable to staff and patients/carers and supported postdischarge communication. Larger trials are needed to establish robust evidence of readmission reduction and error/harm reduction, equity and cost-benefit.

**WHAT IS ALREADY KNOWN ON THIS TOPIC**

- Few tools or resources for postdischarge care of older people in general practice exist, especially those which empower patients and their carers.

**WHAT THIS STUDY ADDS**

- We piloted a new codesigned patient-/carer-led intervention for postdischarge care in general practice and found it to be acceptable to patients and staff.

**HOW THIS STUDY MIGHT AFFECT RESEARCH, PRACTICE OR POLICY**

- The intervention appears to have the potential to make meaningful changes to the quality and safety of patient care. A full implementation package needs to be coproduced to enhance its feasibility for practices.

## Introduction

 Older people are at heightened risk of healthcare-related harm following discharge from hospital due to failures in communication, medication management and follow-up contributing to avoidable morbidity and distress.^1^,^4^ Older patients often report distress and difficulty managing care postdischarge.^5^ They and their informal carers frequently report uncertainty about self-management, unmet information needs and difficulty navigating postdischarge care.^5^,^9^ Although national policy emphasises safe discharge and coordinated follow-up,^10 11^ postdischarge communication in general practice remains under-researched, and a structured, standardised approach to supporting older patients during this vulnerable period is lacking.^12^

Medication-related harm after discharge is well recognised,^13 14^ yet errors involving test requests, follow-up and other aspects of care are also prevalent and less well studied.^1 4^ Timely primary care involvement can reduce readmissions and improve safety,^15^,^17^ yet communication failures remain common.^1 4 13 14^ Many older patients struggle to articulate concerns in standard General Practice appointments, especially when recovering from acute illness or experiencing cognitive or communication difficulties.^18 19^ In a small Australian study, older patients reported an inability to ask questions, especially about potential vital red flags (changes that might indicate clinically concerning deterioration) in the predischarge hospital environment and difficulties fitting their postdischarge concerns into a standard GP appointment.^19^ As hospital stays shorten and complexity shifts into the community, the burden on general practice to manage postdischarge risk continues to grow.

Our previous works show that general practice is uniquely placed to address problems affecting postdischarge care in older patients.^20^ International evidence suggests that structured primary care involvement can reduce harm. A 2022 study found that an early GP appointment postdischarge was associated with substantially lower readmission risk,^15^ and recent US evidence^16^ suggests that a timely primary care provider visit can reduce hospital readmission for adults of all ages. A study of high-risk older people highlighted the need for timely access to primary care to prevent readmissions.^17^ In the UK, there is also confusion among discharged patients about the services existing in community pharmacies,^18^ despite medication reconciliation being a major component of postdischarge care.

This pilot was conducted within the General Practice Management After Transition Events (GP-MATE) study,^21^ which aimed to address gaps in postdischarge communication for older patients and their carers. Codesigned with patients, carers and practice staff,^22^ the GP-MATE intervention (box 1) provides a structured, patient-held communication tool (online supplemental appendix 1) and a dedicated 20-min postdischarge general practice appointment. While earlier phases of the study identified the nature of postdischarge problems and informed intervention development,^21 23^ the purpose of the pilot was to explore the implementation of GP-MATE delivery in routine practice.

Box 1 **GP-MATE intervention components**

**Patient-facing components:**

- Telephone call from a practice administrator to explain GP-MATE and book a dedicated 20-min postdischarge GP-MATE consultation within 14 days of hospital discharge.
- An intervention pack containing:
  - Two welcome letters on practice letterhead (one for patients and another for carers).
  - A paper copy of a 5-page patient-held GP-MATE* (available here) with instructions to complete prior to the GP-MATE consultation.

**Practice-facing components**

- Bespoke 1-to-1 receptionist training on conducting rolling searches to identify recently discharged patients and booking GP-MATE consultations.
- 1-hour group session at each practice for all clinicians delivering GP-MATE consultations.
- Electronic copy of the GP-MATE staff handbook to support delivery (online supplemental appendix 7).

*The information patients/carers provide on their patient-held GP-MATE is part of the intervention and is the starting point for the GP-MATE consultation. GP-MATE, General Practice Management After Transition Events.

This mixed-methods pilot, therefore, aimed to assess feasibility (recruitment, tool use), acceptability (staff/patient views), potential outcomes (error, harm, readmission) and healthcare cost implications.

The findings will inform refinement of the intervention and its implementation strategy ahead of a future trial.

## Methods

### Study design and setting

We conducted a convergent mixed-methods pilot of the GP-MATE intervention in seven general practices in the West Midlands, England. The practices had previously participated in an ethnographic study of postdischarge processes,^23^ in which purposive sampling of practices was used to reflect variation in area, size, rurality, socioeconomic status and ethnic diversity (table 1). Data were collected from multiple sources and analysed convergently from anonymised electronic health records (EHRs) of patients in the intervention cohort, copies of patient-held forms, interviews with patients/carers and staff and patients/carers survey data. The pilot ran from July 2024 to May 2025 and followed the published protocol.^21^ The study was led by an academic GP who participated in all data collection and analysis. The rest of the methods section follows a narrative structure which corresponds to the order of data collection in practices.

**Table 1:** Practice demographics

Practice study code	Practice list size*	Patient population aged 65, %†	Ethnicity (% White)†	Patients per WTE GP†	IMD decile†	Rural/urban†	CQC rating‡	Number of participants recruited
10	12 300	14.8	74.1	1065	2	Urban	Good	7
11	21 000	15.4	68.7	1646	2	Urban	Outstanding	5
13	26 500	5.6	57.3	2470	2	Urban	Good	18
14	8500	18.7	83.3	1758	3	Urban	Good	3
16	12 400	26.8	91.9	883	10	Semi-rural	Outstanding	0
17	8000	19.2	88.8	909	8	Rural	Good	1
18	5700	30.0	93.4	1145	8	Rural	Good	6

Practice study codes correspond to those used in our ethnographic study;^23^ missing numbers occur where rerecruitment to this stage of the study failed.

* Data from practice records; practice list sizes rounded to the nearest 100 to preserve anonymity.

† 2025 data from General Practice Workforce—NHS England Digital.

‡ Data from CQC website: https://www.cqc.org.uk/search/all?filters%5B%5D=services%3Adoctor&radius=all.

CQC, Care Quality Commission; IMD, Index of Median Deprivation; WTE, Whole Time Equivalent.

### Practice recruitment and preparation

At site initiation visits, the research team reviewed each practice’s discharge summary handling processes and agreed local implementation plans. Practices chose whether GP-MATE appointments were delivered face-to-face, by telephone or via home visit and selected the clinicians they felt most appropriate (GPs or pharmacists). Administrators received one-to-one training on conducting weekly rolling searches to identify all recently discharged patients aged ≥65 and book appointments within 2 weeks. Clinicians were trained to use GP-MATE in a one-off, hour-long session at each practice. Follow-up mini interviews with practice administrators were conducted to support intervention implementation and fidelity approximately 1 month into recruitment. To ensure patient safety, practices maintained their usual (reactive) offer for postdischarge care alongside GP-MATE.

### Participant inclusion criteria

Eligible patients were those aged ≥65 who had been discharged from the hospital and attended a GP-MATE appointment. Patients with cognitive impairment were included with carer support. Abbreviated EHR data were also collected for patients who did not attend (DNA) a booked appointment. Clinicians delivering GP-MATE and administrators involved in booking appointments were eligible for the interview. Survey forms (online supplemental appendix 2) and expressions of interest (EOIs) for the interview were returned by self-addressed envelope from patients’/carers’ intervention pack.

### Data sources and collection

#### Four data sources were used

##### Electronic health records (EHRs)

The core study team (an academic GP and a research fellow) extracted anonymised data from the practices’ EHRs on demographics, admission characteristics, care utilisation, discharge summary actions, error/harm and GP-MATE component use. These items mirrored those used in a comparator cohort from the same practices.^4^ EHR data were collected across the pilot, but always more than 90 days after a patient’s GP-MATE consultation. Data were collected without individual consent under appropriate approvals and anonymised before leaving the sites. Data quality checks were undertaken by the core research team throughout the extraction process. Error and harm events were reviewed and agreed through wider research team discussion for each case.

##### Patient-held GP-MATE forms

Forms were retrieved from EHRs (when they had been scanned into the records by practice teams) or volunteered by patients during interviews. Free-text entries were analysed for completeness and content.

##### Patient/carer surveys

Surveys included Likert-scale items and brief free-text responses (online supplemental appendix 2). Patients/carers were encouraged to return surveys soon after GP-MATE consultations. Returned surveys were matched to EHR data once this was collected.

##### Interviews

Brief interviews (around 15 min) with administrators (online supplemental appendix 3) were conducted and analysed early in implementation to explore feasibility and fidelity and to troubleshoot problems with recruitment; these were documented with field notes rather than audio recorded. Semistructured interviews (up to 40 min duration) were conducted with patients/carers in their homes shortly after the GP-MATE appointment and with healthcare practitioners (HCPs) (up to 60 min in duration via Teams or in-practice) towards the end of the pilot (see onlinesupplemental appendices 4  5 for interview schedules). Patient and staff interviews were audio-recorded, transcribed, and anonymised.

### Data analysis

Quantitative data were managed in Microsoft Excel. Descriptive statistics and modelling were conducted in *R*. Healthcare service use was costed using standard National Health Service (NHS) reference sources.^24^ Total, median and mean costs were reported by service type. Survey data were summarised using simple descriptive statistics; due to paucity of free-text responses, these were not subject to a formal qualitative analysis. Transcripts were managed in *NVivo (V15*). Interviews, field notes and patient-held forms were coded using a simple practical codebook suitable for the evaluation of a pilot study. Two researchers (RS and NK) coded transcripts independently, with discrepancies resolved through discussion. Analysis of all data sources was led by RS and occurred concurrently throughout the study and until 6 months after recruitment closed. Framework analysis was used to synthesise the qualitative findings across data sources, with attention to triangulation, silence (where a group does not report on an issue) and dissonance (where participants disagree).^25^

### Patient and public involvement

GP-MATE was codesigned with over 20 Patient Public Involvement (PPI) contributors nationally. Three remunerated core PPI partners supported all stages of the 4-year study, including the development of patient-facing materials and recruitment strategies for the pilot study.

## Results

### Demographics

The seven participating general practice sites are fully described elsewhere;^23^ facsimile table 1 shows their characteristics. Practice 13 recruited 18/40 (45%) of the cohort; site 16 dropped out without recruiting any patients.

Across the practices, 84 patients were contactable by phone and had a GP-MATE appointment booked. Of these, 28 were cancelled and 16 were DNA. There were no differences in ethnicity or gender among participants, but DNAs were slightly older (mean age 77). Two of the DNAs did so because they had been readmitted to the hospital. Non-participants offered a variety of reasons for declining, and 52% (23/44) were not seen by a clinician in relation to their discharge; four died within the 3-month record review period (9% death rate).

The final cohort (table 2) comprised 40 patients with a mean age of 75 years (65–91); 21 were female (53%), eight (20%) had a recorded carer, and one patient died during the data collection period. Most admissions were emergency (72.5%; n=29) and to medical specialties (60%; n=16). The median hospital stay was 9 days (range 1–125 days). Surveys were returned by 29 participants (72.5%) and were matched to EHR data. 17 patient-held GP-MATE forms (42.5%) were retrieved. Seven patient/carer interviews were conducted from 16 EOIs (attrition primarily due to ill health). Nine interviews were conducted from the pool of 10 HCPs who had conducted one or more GP-MATE appointments (table 3).

**Table 2:** Patient participant demographics

	EHR cohort (n=40)	Interviewees (n=7*)	Survey cohort (n=29)	Patient-held cohort (n=17*)
Age (mean and range)	75.35 (65–91)	72.29 (70–79)	75.48 (65–91)	75.29 (65–90)
Patient/carer status	8			
Has a carer recorded/is a carer	8 (20%)	1 (14%)	4	3
Patient/carer dyad	NA	1 (14%)†	NA	NA
Patient	NA	5 (71%)	25	10
Missing	NA	NA	NA	4
Gender†				
Female	21 (53%)	5 (71%)	13 (45%)	9 (53%)
Male	19 (47%)	2 (29%)	16 (55%)	8 (47%)
Ethnicity				
White British	33 (82.5%)	7 (100%)	22 (76%)	14 (82%)
White Irish	1 (2.5%)	0	0	0
Asian	1 (2.5%)	0	1 (3%)	1 (6%)
Missing	5 (12.5%)	0	6 (20%)	2 (12%)
Discharge diagnosis				
Medical	24 (60%)	4 (57%)	15 (52%)	6 (35%)
Surgical	16 (40%)	3 (43%)	11 (38%)	8 (47%)
Specialty missing	0	0	3 (10%)	3 (18%)
Elective	11 (27.5%)	3 (43%)	8 (28%)	7 (41%)
Emergency	29 (72.5%)	4 (57%)	18 (62%)	7 (41%)
Acuity missing	0	0	3 (10%)	3 (18%)
Informal carer on medical record				
Yes	8 (20%)	1 (14%)	4 (14%)	2 (12%)
No	32 (80%)	6 (86%)	22 (76%)	12 (70%)
Missing	0	0	3 (10%)	3 (18%)
Mean length of admission in days (range)	13.8 (1–125)	5.29 (1–12)	17 (1–125)	11 (1–50)

Patient participant demographics for the different data sources. Individual patient/carer demographics are not reported to protect anonymity.

* Two patients contributed patient-held forms but did not have a GP-MATE appointment, and one patient was interviewed but did not have a GP-MATE appointment (cancelled by the patient or practice). We have included their data as they took part in some elements of the GP-MATE process.

† Gender is ascribed to the patient in dyadic interviews, and they are counted as one participant.

EHR, electronic health record; GP-MATE, General Practice Management After Transition Events.

**Table 3:** HCP interviewee demographics

Participant number	Gender	Ethnicity	Professional role	Years of experience in the role
13GP1	Female	Asian Indian	GP	0.5
13GP2	Female	Mixed White	GP	5
13GP3	Female	British Pakistani	GP	11
11P1	Male	Indian	Pharmacist	9
11P2	Male	British Asian	Pharmacist	20
10GP1	Female	White British	GP	25
10GP2	Female	Mixed White Asian	GP	12
17GP	Female	Mixed British	GP	2
18GP	Male	South Asian	GP	20

HCP, healthcare practitioner.

### GP-MATE appointment descriptive data

A GP-MATE appointment was documented for 39/40 participants (one presumed recording omission). The median interval between discharge and GP-MATE appointment was 19 days (range: 5–78 days); only eight (20%) were within the 14-day target. Most (35/39, 90%) appointments were conducted by GPs face-to-face (five at home visits and three by phone), with a small number delivered by pharmacists.

### Use of the patient-held GP-MATE tool

Completion rates on patient-held forms exceeded 70% for all assessed items in the tool (online supplemental appendix 6). Of our two continuity items, patients consistently recorded clinician names, but very few completed the clinician’s working days. Sections often left blank by patients (*medications/red flags*) were sometimes completed by the clinicians, as evidenced by a change of handwriting and ‘medical language’. Despite the importance in the codesign^22^ and 100% completion of the question: “Do you want to talk about loneliness?”, no participant indicated a wish to speak on this matter.

Coverage of GP-MATE domains, as documented in the EHR, varied, with *Information power* and *Medications* being addressed in most appointments (table 4), whereas only 6/40 (15%) covered all four areas. Nevertheless, GP-MATE appointments generated 103 documented changes to care (mean 2.6 changes per appointment), including medication adjustments, referrals, follow-up arrangements and updates to carer information.

**Table 4:** GP-MATE domain coverage within appointments, and resulting changes made to care

GP-MATE domain	Coverage	Coverage (%)	Number of changes made	Changes made (as a % of cohort)	Example of change wrought
Continuity	9/40	23%	6	15%	Confirmatory SMS with appointment details, working days of healthcare practitioner (HCP) volunteered
Carers and caring	19/40	48%	16	40%	Carers' details updated in records and referred to the social prescriber
Medications	27/40	68%	21	52.5%	Medications changed, medication review booked, pharmacist involved in care
Information power	37/40	93%	35	87.5%	Copy of discharge summary provided by the GP, missing follow-up care arranged
Other topic (as directed by patients)	28/40	70%	25	62.5%	Referrals made, investigations ordered and allied health professional appointments made

GP-MATE, General Practice Management After Transition Events; HCP, healthcare practitioner; SMS, text message.

### Care utilisation and cost

Many postdischarge care episodes were evident (see tables5  6 for appointment mode and HCP). Prior to their GP-MATE appointment, 24/40 (60%) of the cohort had already consulted an HCP at the practice *about their admission* (attributable contact), and half of these had more than one prior contact. Across the 3-month follow-up, 115 attributable HCP contacts were recorded for 33 patients, most commonly by telephone (57%) or face-to-face (33%).

**Table 5:** Mode of attributable general practice contact

	Telephone	FTF	Home visit	SMS
Number	66	38	9	2
%	57.4	33.0	7.8	1.74

Attributable contacts n=115 (which is the denominator).

FTF, face-to- face; SMS, text message.

**Table 6:** Healthcare professional (HCP) undertaking attributable general practice contact

	GP	Pharmacist in general practice	AHP	Nurse	Non-clinical
Number	48	27	17	13	10
%	41.74	23.5	14.8	11.3	8.7

Attributable contacts n=115 (which is the denominator).

AHP, allied health professional; FTF, face-to- face.

The median attributable HCP cost per patient was £160.24 per user, with a mean cost of £180.71 (SD £81), including the £98 GP-MATE appointment. A similar mean cost of £89.75 (SD £69.37) was observed in our comparator cohort.^4^ Most patients (28/40) had attributable care only in primary care. 12 patients required urgent or emergency care, with highly skewed costs due to a small number of frequent reattenders. For these patients, the median cost of attributable urgent and emergency care utilisation was £1107.66 per patient (n=12), with a mean cost £2509.51 (SD £4284.52). One patient was readmitted five times at a cost exceeding £13 000.

### Error and harm

There were 151 actions (defined as required changes to care as stated in the discharge summary) for 31 patients. Two patients (2/31) experienced errors (failure to complete actions without a documented reason), both involving failure to initiate new medications (bone protection and a laxative). Error rates were lower than in the comparator cohort (20.6%)^4^ and significantly lower than earlier 2016 data for patients aged ≥75 years (13.3% compared with 46%; p=0.01), although the pilot was underpowered for definitive conclusions. For the two patients where an error occurred, the clinician reviewer searched the EHR for related harms (eg, re-presentation with constipation or Urinary Tract Infection where a laxative was not prescribed). No harms were detected (0/31=0%, 95% CI 0% to 11.1%), but due to low harm rates in our comparator cohort of 4.4%^4^ and 2016 data, 8%,^1^ this study was not significantly powered to demonstrate statistically significant differences. Multivariate modelling for error/harm was not undertaken, as no covariates were statistically significant and perfect separation occurred for several variables.

### Process evaluation

We report our survey, interview and free-text data from patient-held forms interwoven for a holistic sense of how the intervention was perceived according to: experiences, usefulness, acceptability/feasibility and barriers/adaptations.

### Experiences of GP-MATE

Overall responses to GP-MATE were positive. 59% (17/29) of survey respondents reported that the GP-MATE process improved their communication with their general practice, and only one person disliked the GP-MATE process overall. Most patient interviewees indicated moderate or high positivity.

It’s like giving you an extra leg … and giving them [GPs] an awareness of what’s happening out of sight. (A female in her 60s, elective orthopaedic)

HCPs mostly described positive experiences, valuing the structured opportunity to address postdischarge questions and undertake safety checks:

a lot of patients have a lot of questions afterwards. A lot of things can be missed out and things, and I found that it was just nice to just get a, kind of, holistic kind of thing at the end, after the discharge… I think patients appreciated it a little bit, just having that little bit longer time with the clinician, going through some of the questions (13GP3).

A minority of patients, particularly those who were unwell or cognitively impaired, struggled to distinguish GP-MATE from usual care. One patient had limited recall of their appointment; another reported feeling rushed and unable to perceive its value.

She [the GP] says, 'I'm sorry…we're only allowed so many minutes…' I thought, 'Well…I thought you were going to sort me out a little bit. (A female in her 70s, heart disease)

Use of the prototype HCP toolkit (online supplemental appendix 7) was low. Most HCPs felt confident to undertake GP-MATE appointments following training and did not require the toolkit, although one reported using the toolkit to positive effect.

### Perceived usefulness

Most patients and carers described the GP-MATE appointment as reassuring, offering improved access and continuity.

It was a face-to-face with the GP, and fortunately one of the few we know there”…. “You get the relationship with your doctor that you used to get years ago. (A female in her 80’s, constipation)

A stipulation for practices in the pilot was that they would offer a dedicated appointment, thereby ensuring access, which patients appreciated:

"it also gave me a chance to actually see a doctor, which was valuable for me."…“If I was to phone up tomorrow, I’d just be any other person phoning up” (a male in his 70s, elective orthopaedic).

HCPs reported that the tool facilitated smoother conversations, especially around medications, and in some cases reduced subsequent workload by clarifying issues early or enabling opportunistic monitoring (eg, blood pressure). Pharmacists commented, in particular, about the fluidity of appointments:

it was a much smoother process, because they have all that information there in writing. They've, kind of, consciously been thinking about their medication post-discharge already. So, it is a much smoother conversation to facilitate. (11P1)

Benefits to the practice (particularly in efficiency gains) were noted:

I hope that the explanation around medication would stand us in good stead going forward, in terms of reducing workload in terms of promoting the, the patient to take control and ownership of, of their management. (10GP2)

If one thing is clarified, we can cut at least some four or five phone calls to the reception. (18GP)

Some GPs who had initially been reticent about the utility of scanning the patient-held GP-MATE onto the EHR had come to appreciate the novelty of the information recorded in patient-held forms:

It is really helpful if we have a lasting power of attorney recorded. (10GP1)

Direct benefits for patients were commonly cited by HCPs and patients themselves, often these centred on clarification of medication reconciliation:

There was an example of a patient who had been admitted with kidney problems who had had a acute kidney injury and majority of their medications were, were stopped…he was completely confused initially, but from, from that appointment, he was able to, kind of, understand why they were all changed and the monitoring that was required. (11P2)

(I) Questioned if still needed Carbocisteine (1111 patient-held form).

Referrals or additional services were also often arranged or clarified:

a lot of the time, they did want social prescribing, or something and then I would do a referral on the back of that. (13GP1)

Awaiting follow up in urology after BCG infusion *(bladder cancer treatment)* is available (GP has added to 1105 patient-held form).

Health literacy benefits for their patients were frequently noted by staff:

one brought a carer in, her carer daughter in with her, and they had got a lot of questions to ask about the discharge summary, things that hadn't been included on it that they've been told in hospital. So they had lots of stuff to, to ask. They hadn't documented it on here, but asking around the questions seemed to work (10GP1).

some of them were so ill during the hospital admission that they actually didn't know what was going on…Quite a few of the patients didn't, wouldn't have read their discharge summary. They didn't know what medication they were supposed to be taking (13GP1).

Although patients did not explicitly refer to their own ‘health-literacy’, comments relating to improved understanding were common:

I had a letter from the hospital and I wanted the doctor to look at it to explain to me what she thought. And she explained to me what the hospital had put and what it meant to me. (1708 patient-held form)

Red flags were not a theme from the interview data, but patient-held forms sometimes contained information on red flags written by patients/carers after discussion with HCPs, suggesting increased health literacy, for example.:

Just swelling in leg and pain relief (1703 patient-held form).

More often, red-flag information was added by HCPs for patients to take away (as evidenced by change in handwriting), for example,

Burning sensation, blood in urine, discharge, red, swelling, temperature (1105 patient-held form).

Patients who felt generally well (especially after elective admissions) identified a ceiling effect with the usefulness of GP-MATE and while this was echoed by HCPs, it was recognised that it is often difficult to anticipate which patients will benefit:

I think some patients would find it incredibly beneficial. I think some patients would just find it an extra admin burden that they wouldn't want to engage with. And it would be very difficult to figure out beforehand which of them it would be. (17GP)

### Acceptability and feasibility (including barriers and adaptations)

GP-MATE was considered acceptable by both patients and staff. The double appointment times were appreciated by HCPs, particularly for patients with medical complexity, although this was not specifically commented on by patients/carers. Practices found the double slots were sometimes hard to book in a timely manner. Most HCPs felt it was feasible to incorporate GP-MATE with sufficient funding and time resource:

Previously, my mind, my mindset was that, like, "Why should I waste my time on explaining things which are not done properly by the hospital?" Now, my mindset is more about why this patient is unnecessarily suffering, due to the inadequacies of the system. (18GP)

A discordant view was obtained from one GP:

It, it felt, actually, a little bit of an extra kind of thing to do, rather than a tool to help me (13GP2).

However, this same GP also felt that GP-MATE was acceptable to patients:

it seemed that the family were a little bit more involved…It seemed that everybody was around, and, kind of, interested, 'cause this was a thing, a special thing. Whereas without the GP-MATE, it would just be me going, doing the home visit, perhaps. (13GP2)

Our survey found that most patients/carers (62%) were confident completing the patient-held GP-MATE form, and 70% reported it was little or no effort to do so. Nevertheless, some participants who were interviewed—particularly those who were unwell/medically complex—found the GP-MATE patient-held form to be challenging to complete; 8/17 patients declared requiring help to fill it out, mainly from carers. Patients universally indicated that the typesetting of the form left adequate space for them to fill out their concerns. Patients/carers did not consider anything other than the use of a paper-based GP-MATE, but HCPs frequently raised the digitisation of GP-MATE. Some favoured delivery to patients via SMS templates that download directly into the EHR, but others feared digital exclusion. It was also felt that a paper-based record was useful in the context of an in-person appointment:

…for you to, to fill out with them when, you know, during the face-to-face appointment. (11P1)

HCPs found the patient-held tool to be variably completed, the medicines table especially poorly so, though HCPs stressed the value of a complete table to them. HCP perception was also that support from a carer increased engagement. HCPs identified a need for preappointment support to have patients fill out the patient-held form, often suggesting a phone call with a care navigator or pharmacy technician to ensure patients understood the GP-MATE tool and reminded them to bring it with them.

If they had filled [it] in properly, if they had done what was expected from that form, it would have been brilliant. (18GP)

Administrators struggled with operational issues in relation to booking appointments for a GP-MATE appointment. They identified barriers in recruiting patients who were housebound, confused, had low health literacy or generally felt unwell, partly due to their own ability to offer and partly due to patient reticence to accept the GP-MATE appointment. Low uptake meant allocated slots sometimes went unused. The read-time burden of accessory study information was commonly cited by administrative staff as off-putting:

Thinks some who DNA’d got cold feet when they saw the pack. (field notes practice 11)

Cancellation of appointments or non-attendance (DNA) rates were high, even at sites with the highest recruitment of patients:

Eight of seventeen booked patients cancelled … reasons unknown as cancellations taken by reception. (field notes practice 10)

Practice 16 withdrew prior to recruiting any patients citing high DNA rates, unfortunately without making non-participant data available to us. At sites where the key administrator changed during the course of the pilot, recruitment at the site collapsed, showing how key these motivated non-clinical staff were to the GP-MATE process. Administrators reported that the offer of a particular HCP improved the uptake of GP-MATE, making the appointment feel ‘special’.

## Discussion

### Summary of main findings

This pilot demonstrates that GP-MATE is feasible to deliver in routine general practice and can surface important gaps in postdischarge communication and care. Despite 60% of patients in the pilot having already had postdischarge care in general practice, GP-MATE appointments still generated clinically meaningful actions, particularly relating to medication reconciliation, follow-up and clarifying discharge information. There was tentative evidence that GP-MATE led to safer care, with lower error rates than previous cohorts,^4^ although the study was underpowered to draw definitive conclusions regarding safety outcomes. Qualitative findings indicate strong acceptability among patients and clinicians who take up GP-MATE, with the personalised, structured format perceived as valuable even when prior contact had occurred. As a multipart intervention, GP-MATE proved complex to implement, and several operational barriers were identified, particularly within administrative processes.

### Strengths and limitations

A particular strength was the extensive codesign process underpinning GP-MATE, which involved practices, patients and carers, ensuring that the intervention was relevant to real-world primary care contexts. This likely contributed to high engagement with patient/carer interviews, despite a potentially frail and unwell cohort, and survey completion rates (72.5%), which exceeded typical response rates in similar populations.^26^ Sustained engagement with the participating practices over 4 years allowed trust and familiarity to develop between researchers and practices, enabling deeper insight into implementation challenges. Detailed review of EHR free-text and document attachments enabled confident identification of discharge-related actions and errors and linkages to downstream events, such as readmissions, harms and healthcare costs. Convergently analysing this data with our linked qualitative datasets provided additional insights into the quality of care at discharge.

However, patient recruitment was lower than anticipated, reflecting known challenges in general practice research^27^ compounded by the cross-system nature of postdischarge enquiry. This limited the scope for triangulation with patient/carer interviewee data, even though EOI was good as a percentage (16/40, 40%). The inclusion of other patient-generated data sources in the triangulation (survey, patient-held forms) mitigated this somewhat. In addition, a localised failure in the hospital IT system responsible for sending discharge summaries to our study practices (as reported extensively nationally^28^), halted patient recruitment for four of the nine study months. Funder restrictions prevented a study pause or extension, which, together with the study requirement for 90 days postdischarge data, severely limited the sample size and scope for statistical modelling and interpretation of economic data. Protocol fidelity was also variable; although GP-MATE was designed to be delivered within 2 weeks of discharge, the median time to appointment was 19 days, reflecting operational pressures and lack of system integration. The cohort lacked ethnic diversity despite practice selection in diverse areas, and the appointment process may have unintentionally favoured patients who were well, English-speaking, more able to engage or willing to participate in a research study, leading to unintentional selection bias. There were some indications that non-participants were either sicker or less likely to attend the practice for care; paradoxically, the patients who are most likely to benefit from the GP-MATE approach, and this calls for alternative implementation approaches. Other causes of non-attendance, such as perception barriers, for example, misunderstanding the purpose of GP-MATE or lack of trust, need further exploration in future studies.

### Comparison with other literature

This study represents a first step in implementing a much-needed context/systems-based primary care complex intervention,^29^ where implementation in this setting is known to be challenging.^30^ The findings align with international evidence that structured healthcare involvement after discharge can reduce harm and improve continuity. Patient-filled transition interventions have been extensively studied in US secondary care^31^ but are little-used in primary care. Actions generated from GP-MATE were often multiple and demonstrated unmet care needs, aligning with expressed needs in other studies.^8 9^ As the number of older patients discharged from hospital grows and capacity in general practice remains limited, consideration of risk-stratified enhanced access routes is warranted (and this may mitigate against the ceiling effects seen in both our quantitative and qualitative data). Automated risk assessment for GP home visiting of older patients after discharge has been trialled with success in Australian systems.^32^ Stratification by condition for older populations is one approach; for example, early GP review following discharge for heart failure has received much attention^33 34^ and may even reduce the risk of death.^35^

### Implications for policy, practice and research

At policy level, the GP-MATE study highlights the need for structured, resourced postdischarge communication pathways in primary care, particularly for older patients at risk of poor-quality transitions.^23^ However, the challenges practices experienced emphasise the need for commissioners and policy makers to carefully consider how its implementation can be supported and funded. Results from our pilot were used to codesign our further implementation strategy at a remunerated national-level day-long workshop with PPI and HCPs in June 2025.

For general practice, the study demonstrates that while clinicians found the intervention acceptable and useful, administrative processes around recruitment and booking were the main barriers; investment in care navigators or digital tools could enhance feasibility. We have shown that postdischarge care for those 65 and over in general practice occurs frequently and starts early;^4^ a practical next step could be to provide tools like GP-MATE at the point of hospital discharge so patients/carers can use it at these index appointments. For this to be effective, all GP sites in a regional study area must be ready to engage with the approach. Engagement of primary care leaders with this approach to shared care across the interface will be engendered by increasing the understanding of the primary care time and resources already being devoted to this cohort. Although at increased risk postdischarge,^4^ no care home residents participated in GP-MATE. This may be because most practices would not offer GP-MATE home visits, which could also, in part, explain high DNA rates. This reluctance reflects financial and practical barriers: home visits take longer than 20 min and involve unremunerated travel costs (often borne personally by HCPs). If GP-MATE is to help those most at risk (the housebound frail), we need to find ways to fight against the tide of home visit delegation^36^ or research how visiting community services (who have a significant impact on readmissions^37^) could deliver the intervention.

Care needs to be taken that the GP-MATE approach does not deepen social inequality^38^ by providing preferential access to those who already have the means to engage with healthcare.^39^ Carefully designed trials of the GP-MATE approach are therefore warranted to further understand how and where it can bring the greatest benefit to the newly discharged older population. Implementation and cost-benefit issues should be fully considered in this work to progress knowledge mobilisation. Future research should test GP-MATE at scale, with diverse patient populations, and evaluate its impact on clinical outcomes such as medication safety, patient empowerment and hospital readmissions. Trials should also explore digital adaptations alongside paper-based tools to balance accessibility with efficiency.

## Conclusion

GP-MATE is a promising, codesigned intervention that supports safer and more coherent postdischarge care. While feasible and acceptable, delivery through general practice alone was challenging, and its implementation package needs further refinement in relation to administrative processes and equitable access. This pilot partially met its objectives in validating the GP-MATE intervention and generated important insights to inform a future trial. The GP-MATE tool is freely available for wider use and further evaluation.^22^

## Supplementary material

10.1136/fmch-2026-003981 online supplemental appendix 1

10.1136/fmch-2026-003981 online supplemental appendix 2

10.1136/fmch-2026-003981 online supplemental appendix 3

10.1136/fmch-2026-003981 online supplemental appendix 4

10.1136/fmch-2026-003981 online supplemental appendix 5

10.1136/fmch-2026-003981 online supplemental appendix 6

10.1136/fmch-2026-003981 online supplemental appendix 7

## Data Availability

Data are available upon reasonable request.

## References

[1] Spencer RA, Spencer SEF, Rodgers S (2018). Processing of discharge summaries in general practice: a retrospective record review. Br J Gen Pract.

[2] Denholm R, Morris R, Purdy S (2020). Impact of emergency hospital admissions on patterns of primary care prescribing: a retrospective cohort analysis of electronic records in England. Br J Gen Pract.

[3] Williams H, Edwards A, Hibbert P (2015). Harms from discharge to primary care: mixed methods analysis of incident reports. Br J Gen Pract.

[4] Klepacz N, Long A, Shariff Z (2026). General practice follow-up after hospital discharge in older adults: Retrospective records analysis. Br J Gen Pract.

[5] Ford DM, Budworth L, Lawton R (2023). In-hospital stress and patient outcomes: A systematic review and meta-analysis. PLoS One.

[6] Coatsworth-Puspoky R, Dahlke S, Duggleby W (2022). Older persons with multiple chronic conditions’ experiences of unplanned readmission: An integrative review. Int J Older People Nurs.

[7] Healthwatch (2025). From hospital to home: improving patient discharge. https://www.healthwatch.co.uk/news/2025-02-04/hospital-home-improving-patient-discharge.

[8] Holland DE, Mistiaen P, Bowles KH (2011). Problems and unmet needs of patients discharged “home to self-care”. Prof Case Manag.

[9] McCusker J, Yaffe M, Lambert SD (2020). Unmet needs of family caregivers of hospitalized older adults preparing for discharge home. Chronic Illn.

[10] Department of Health & Social Care (2024). Hospital discharge and community support guidance. https://www.gov.uk/government/publications/hospital-discharge-and-community-support-guidance/hospital-discharge-and-community-support-guidance.

[11] National Institute for Health and Care Excellence (2015). Transition between inpatient hospital settings and community or care home settings for adults with social care needs [NG27]. NICE guideline. https://www.nice.org.uk/guidance/ng27/resources/transition-between-inpatient-hospital-settings-and-community-or-care-home-settings-for-adults-with-social-care-needs-pdf-1837336935877.

[12] Spencer RA, Singh Punia H (2021). A scoping review of communication tools applicable to patients and their primary care providers after discharge from hospital. Patient Educ Couns.

[13] Parekh N, Ali K, Davies JG (2020). Medication-related harm in older adults following hospital discharge: development and validation of a prediction tool. BMJ Qual Saf.

[14] Alqenae FA, Steinke D, Keers RN (2020). Prevalence and Nature of Medication Errors and Medication-Related Harm Following Discharge from Hospital to Community Settings: A Systematic Review. Drug Saf.

[15] John G, Payrard L, Donzé J (2022). Associations between post-discharge medical consultations and 30-day unplanned hospital readmission: A prospective observational cohort study. Eur J Intern Med.

[16] Kojima N, Bolano M, Sorensen A (2022). Cohort design to assess the association between post-hospital primary care physician follow-up visits and hospital readmissions. Medicine (Baltimore).

[17] Antony SM, Grau LE, Brienza RS (2018). Qualitative study of perspectives concerning recent rehospitalisations among a high-risk cohort of veteran patients in Connecticut, USA. BMJ Open.

[18] Khayyat S, Walters P, Whittlesea C (2021). Patient and public perception and experience of community pharmacy services post-discharge in the UK: a rapid review and qualitative study. BMJ Open.

[19] King L, Harrington A, Nicholls S (2023). Towards reduction of preventable hospital readmission: Older people and family members’ views on planned self-management of care at home. J Clin Nurs.

[20] Spencer RA, Rodgers S, Salema N (2019). Processing discharge summaries in general practice: a qualitative interview study with GPs and practice managers. BJGP Open.

[21] Shariff Z, Dale J, Spencer RA (2024). General practice management after transition events: protocol for an experience-based co-design study. BJGP Open.

[22] Ann Spencer R, Shariff Z, Dale J (2025). Promoting health literacy of older post-discharge patients in general practice - Creation of the GP-MATE communication tool through co-design. Patient Educ Couns.

[23] Spencer RA, Shariff Z, Dale J (2025). Safety issues in post-discharge care of older patients in general practice: an ethnographic study. Br J Gen Pract.

[24] NHS England National cost collection for the NHS 2023/24. https://www.england.nhs.uk/publication/2023-24-national-cost-collection-data-publication/.

[25] Farmer T, Robinson K, Elliott SJ (2006). Developing and implementing a triangulation protocol for qualitative health research. Qual Health Res.

[26] Booker QS, Austin JD, Balasubramanian BA (2021). Survey strategies to increase participant response rates in primary care research studies. Fam Pract.

[27] Peto V, Coulter A, Bond A (1993). Factors affecting general practitioners’ recruitment of patients into a prospective study. Fam Pract.

[28] Pulse Articles on delayed discharge summaries and other hospital correspondence.

[29] May CR, Mair FS, Dowrick CF (2007). Process evaluation for complex interventions in primary care: understanding trials using the normalization process model. BMC Fam Pract.

[30] Lau R, Stevenson F, Ong BN (2015). Achieving change in primary care--effectiveness of strategies for improving implementation of complex interventions: systematic review of reviews. BMJ Open.

[31] Buurman BM, Parlevliet JL, Allore HG (2016). Comprehensive Geriatric Assessment and Transitional Care in Acutely Hospitalized Patients: The Transitional Care Bridge Randomized Clinical Trial. JAMA Intern Med.

[32] Surate Solaligue DE, Hederman L, Martin CM (2014). What weekday? How acute? An analysis of reported planned and unplanned GP visits by older multi-morbid patients in the Patient Journey Record System database. J Eval Clin Pract.

[33] Feltner C, Jones CD, Cené CW (2014). Transitional care interventions to prevent readmissions for persons with heart failure: a systematic review and meta-analysis. Ann Intern Med.

[34] Murtaugh CM, Deb P, Zhu C (2017). Reducing Readmissions among Heart Failure Patients Discharged to Home Health Care: Effectiveness of Early and Intensive Nursing Services and Early Physician Follow-Up. Health Serv Res.

[35] McAlister FA, Youngson E, Bakal JA (2013). Impact of physician continuity on death or urgent readmission after discharge among patients with heart failure. CMAJ.

[36] Abrams R, Wong G, Mahtani KR (2020). Delegating home visits in general practice: a realist review on the impact on GP workload and patient care. Br J Gen Pract.

[37] Coffey A, Leahy-Warren P, Savage E (2019). Interventions to Promote Early Discharge and Avoid Inappropriate Hospital (Re)Admission: A Systematic Review. Int J Environ Res Public Health.

[38] McConnachie A, Ellis DA, Wilson P (2023). Quantifying unmet need in General Practice: a retrospective cohort study of administrative data. BMJ Open.

[39] Levy H, Janke A (2016). Health Literacy and Access to Care. J Health Commun.

