# Supplementary material for: Pilot of a novel patient-led intervention for postdischarge from hospital management of older patients’ care in general practice
Source: Fam Med Community Health. 2026 Jul 8;14(3):e003981. doi: 10.1136/fmch-2026-003981 (PMC13347910; doi:10.1136/fmch-2026-003981)
Supplement: online supplemental appendix 1 [file fmch-14-3-s001.docx]

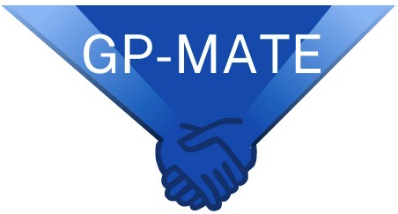


Please fill out ready for your GP-MATE appointment

This is your GP-MATE patient-held record, a space for you to write about your recent hospital admission. We hope you find it a helpful document **both to fill out and to keep**. You should fill it out as soon as you feel able after you come home from hospital and before your GP-MATE appointment.

Not all sections apply to everybody, if a part is not relevant to you then move on. Don’t worry if there are parts you’re not sure about. If these parts are important to you then mark them with a * so they can be addressed at your GP-MATE appointment. With your permission, the information you enter in GP-MATE will be recorded by your GP practice.

Some people filling out GP-MATE will be family or carers acting on behalf of an older person who has come home from hospital. We refer to them as ‘carers’. We’ve tried to make our questions apply whether you are in a caring role or are answering for yourself. It’s OK to fill out GP-MATE with your carer or family member if you want to.

Best wishes for a speedy recovery.

The GP-MATE team

I am filling this out as a patient / carer (please circle)

If you are filling this form out as a carer, where the question states “you” or “your,” please answer for the person you care for.

Name of patient and Date of Birth:

………………………………………………………………………………………………………………………………………………

Name and contact number of main carer: (if relevant)

………………………………………………………………………………………………………………………………………………

Relationship to patient (if relevant):

………………………………………………………………………………………………………………………………………………

Does anybody hold Lasting Power of Attorney* for health and welfare? If so, please state their name and contact details.

……………………………………………………………………………………………………………………………………………………………………………………………………………………

* <https://www.nhs.uk/conditions/social-care-and-support-guide/making-decisions-for-someone-else/giving-someone-power-of-attorney/>

Reason for admission to hospital: …………………………………………………………………………………………………………………………………........................................................................................

What do you hope to get out of your GP-MATE consultation?

………………………………………………………………………………………………………………………………………………………………………………………………………………………


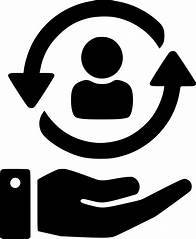
 **Continuity of care after discharge**

My GP-MATE appointment is on:……………………(date) at ………………(time) with: ………………………………………………………………

Days they usually work: ………………………………………………………………………

If you have any urgent symptoms or concerns, please don’t delay getting an appointment with a different practitioner. Staff have multiple roles which may mean you’re not always able to see them on the days they usually work.


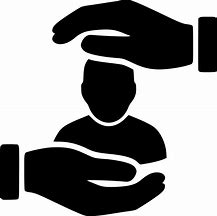
 **Carers and caring after discharge**

1) Is anyone helping you out since you came home from hospital?

Yes / No (circle)

if No, proceed to 2.

1a) Who is/are the key person/people who help you out?

………………………………………………………………………………………………………………………………………………………………………………………………………………………………………………………………………………………………

1b) How do they help you out?

………………………………………………………………………………………………………………………………………………………………………………………………………………………

1c) Are you receiving any medical care at home? (E.g., wound care, district nurse visits)

………………………………………………………………………………………………………………………………………………………………………………………………………………………

1d) Is there anything that you feel is missing from the care that you are currently receiving?

………………………………………………………………………………………………………………………………………………………………………………………………………………………

If you are a carer looking for more information you might find the following organisations helpful:

<https://www.carersuk.org/>

https://www.ageuk.org.uk/

<https://store.redcross.org.uk/pages/categories>

(independent living equipment)

2) Do you live alone? Yes / No

2a) Do you want to talk about loneliness during your GP-MATE consultation?

Yes / No

For support with loneliness try  [Age UK](https://www.ageuk.org.uk/information-advice/health-wellbeing/loneliness/) [The Silver Line Helpline](https://www.thesilverline.org.uk/)

<https://www.befriending.co.uk/directory/>

<https://www.reengage.org.uk>


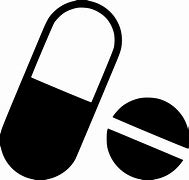
**Medications after discharge**

Your GP or pharmacist will have checked your medications with your discharge letter before your GP-MATE appointment. It’s helpful for us to go through your understanding of medication changes that may have been made and this table will help us to do this. Changes include a new medication being added, a dose change, or a medication being stopped. If you need more space, an extra table can be found at the end of GP-MATE. The following websites might be helpful: Medicines A-Z - NHS (www.nhs.uk) [Find a pharmacy - NHS (www.nhs.uk)](https://www.nhs.uk/service-search/pharmacy/find-a-pharmacy). If there have been no changes, please go to Section 4 (Information Power) (Please * any parts of the table you’re not sure about – your practitioner can go through these with you at your appointment)

Please tick this box if you can’t manage to fill this table out now, but would like to try again later 🗆

| Name of medication | Newly started in hospital (tick) | Changed dose (tick) | Stopped  (tick) | What is/was it for? | Why was it added/changed/stopped? | Temporary (T) or Permanent (P) change? |
| --- | --- | --- | --- | --- | --- | --- |
|  |  |  |  |  |  |  |
|  |  |  |  |  |  |  |
|  |  |  |  |  |  |  |
|  |  |  |  |  |  |  |

3) Is there anything that you are concerned about in relation to your medication since coming home?

*………………………………………………………………………………………………………………………………………………………………………………………………………………………………………………………………………………………………………………………………………………………………………………………………………………………………………………………………………………………………………………………………………………………………………………………………………………………………………………………………………………………………………………………………………………*

Your pharmacist at the chemists may be able to go through **new** medications for some common conditions in more detail, ask for a **“New medicines Service”** check-up. New Medicine Service (NMS) - NHS ([New Medicine Service (NMS) - NHS (www.nhs.uk)](https://www.nhs.uk/nhs-services/prescriptions-and-pharmacies/pharmacies/new-medicine-service-nms/)

**
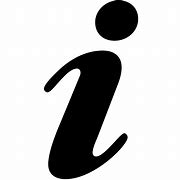
Information power after discharge**

Place a * next to any question you’re not sure about.

4) Do you have a copy of your discharge summary letter from your stay in hospital?

Yes / No (circle)

if No to proceed 4d) below

4a) Is there anything in the discharge letter which you wish to talk about at your GP-MATE appointment?

……………………………………………………………………………………………………………………………………………………………………………………………………………………………………………………………………………………………..

4b) Do you feel there is anything missing from your discharge letter?

………………………………………………………………………………………………………………………………………………………………………………………………………………………………………………………………………………………………

4c) Do you have any questions about the follow up healthcare (e.g., tests, follow-up appointments) that have been described in the discharge letter?

………………………………………………………………………………………………………………………………………………………………………………………………………………………………………………………………………………………………………………………………………………………………………

4d) Is there anything you are concerned about in relation to either your physical or mental health since coming home?

………………………………………………………………………………………………………………………………………………………………………………………………………………………………………………………………………………………………

………………………………………………………………………

4e) What is your health priority after coming home from hospital?

………………………………………………………………………………………………………………………………………………………………………………………………………………………………………………………………………………………………

……………………………………………………………………………………………………………………………………………….

……………………………………………………………………….

Complete this question during or after your GP-MATE appointment:

4f) What does your practitioner want you to keep an eye on?

……………………………………………………………………………………………………………………………………………………………………………………………………………………………………………………………………………………………..

……………………………………………………………………..

Now that you’ve had a chance to go through GP-MATE, please feel free to return to the final question in the introductory section – “What do you hope to get out of your GP-MATE consultation?” to add anything you might have thought about while completing GP-MATE

**Use this space for any notes made during or after the GP-MATE appointment:**

………………………………………………………………………………………………………………………………………………………………………………………………………………………………………………………………………………………………………………………………………………………………………………………………………………………………………………………………………………………………………………………………………………………………………………………………………………………………………………………………………………………………………………………………………………………………………………………………………………………………………………………………………………………………………………………………………………………………………………………………………………………………………………

*This tool is part of the GP-MATE research study and is under development as part of our package of GP-MATE resources. It remains the intellectual property of the University of Warwick. Copyright © November 2023 Rachel Spencer.* Permission granted to reproduce for personal and educational use only. Commercial copying, hiring, lending is prohibited.


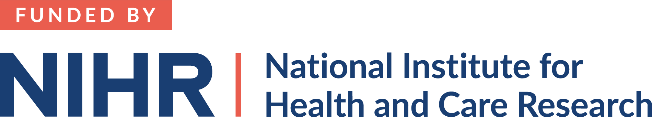


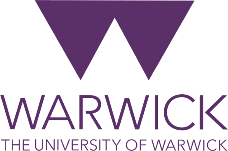


*This study/project is funded by the NIHR Advanced Fellowship 301328. The views expressed are those of the author(s) and not necessarily those of the NIHR or the Department of Health and Social Care*.

Reproduced verbatim under CCBY NC from: Spencer RA, Shariff Z, Dale J. Promoting health literacy of older post-discharge patients in general practice - Creation of the GP-MATE communication tool through co-design. Patient Educ Couns. 2025;130:108474.
