# Supplementary material for: Pilot of a novel patient-led intervention for postdischarge from hospital management of older patients’ care in general practice
Source: Fam Med Community Health. 2026 Jul 8;14(3):e003981. doi: 10.1136/fmch-2026-003981 (PMC13347910; doi:10.1136/fmch-2026-003981)
Supplement: online supplemental appendix 2 [file fmch-14-3-s002.docx]

**Survey for patients who have experienced GP-MATE**

Dear patients and carers,

This survey takes less than 2 minutes to complete. Please fill out this survey after completing your GP-MATE appointment at your practice. If you decided not to take up a GP-MATE appointment, please still fill in the survey to let us know why you didn’t.

Please circle……..

1. Are you a patient or an informal carer?

| Patient | Informal Carer |
| --- | --- |

informal carers are unpaid family or friends who care

1. How confident were you to fill out the GP-MATE patient-held form?

| Very unconfident | Unconfident | No opinion | Confident | Very Confident |
| --- | --- | --- | --- | --- |

1. Did you get any help filling out the GP-MATE patient-held form?

| Yes | No |
| --- | --- |

If yes, who helped: …………………………………………………………………………

1. How much effort did it take to complete the GP-MATE patient-held form?

| Huge effort | A lot of effort | No opinion | A little effort | No effort at all |
| --- | --- | --- | --- | --- |

1. There was enough space to write on the GP-MATE patient-held form.

| Strongly disagree | Disagree | No opinion | Agree | Strongly Agree |
| --- | --- | --- | --- | --- |
|  |  |  |  |  |

**PTO**

1. Did you have a GP-MATE appointment with your general practice?

| Yes | No |
| --- | --- |

1. The GP-MATE approach has improved my communication with the general practice after coming home from hospital.

| Strongly disagree | Disagree | No opinion | Agree | Strongly Agree |
| --- | --- | --- | --- | --- |
|  |  |  |  |  |

1. Using GP-MATE **interfered** with my **other** priorities.

| Strongly disagree | Disagree | No opinion | Agree | Strongly Agree |
| --- | --- | --- | --- | --- |
|  |  |  |  |  |

1. Overall, did you like or dislike GP-MATE?

| Strongly dislike | Dislike | No opinion | Like | Strongly like |
| --- | --- | --- | --- | --- |
|  |  |  |  |  |

Is there anything else you’d like to tell us about GP-MATE or do you have any suggestions for how it can be improved?

…………………………………………………………………………………………………………………………………………………………...........................................................................................................................................................................................................................................................................................................................................................................................................................................................................................
