# Supplementary material for: Pilot of a novel patient-led intervention for postdischarge from hospital management of older patients’ care in general practice
Source: Fam Med Community Health. 2026 Jul 8;14(3):e003981. doi: 10.1136/fmch-2026-003981 (PMC13347910; doi:10.1136/fmch-2026-003981)
Supplement: online supplemental appendix 3 [file fmch-14-3-s003.docx]

GP-MATE Interview with Healthcare Staff Topic Guide

**Project introduction**

Thank you for taking part in the GP-MATE study and offering the consultations with patients. We would like your help to get the design of GP-MATE right. As part of the study your general practice has been using GP-MATE. We are interested in how you used it and what you thought about it. If you didn’t use it, we are interested in the reasons why.

Does that sound clear to you?

Do you have any questions at this stage?

(TAKE WRITTEN CONSENT)

CHECK DEMOGRAPHICS INFORMATION COMPLETE if not captured by email

**How long have you worked at your practice?**

**How many years’ have you been qualified in your current role?**

**Age and ethnicity**

1. Can we talk about the GP-MATE consultations themselves……

**Can you tell me about your personal experience of GP-MATE consultations with patients/carers?**

Prompt the staff to get out the 3 cases prepared for the interview (iterate the following questions across the 3 cases)

**Did you see evidence of patients using written notes on their GP-MATE either before, during or after the consultation?**

If yes,

**Did these written notes influence the consultation? In what way?**

**Did your care of patients change as a result of using GP-MATE?**

If yes, prompt in what ways? : medications, follow-up, social care, health literacy, trust

**Were there any positives for you as a practitioner using GP-MATE?**

**Were there any negatives for you as a practitioner using GP-MATE?**

**Were there any benefits to patient/carer who used GP-MATE?**

**Were there any disadvantages to patient/carer who used GP-MATE?**

**Were you able to complete the GP-MATE consultations in the 20 minute slot?**

If not, why not? (prompts – complex patients, features of the GP-MATE patient held tool)

**Did you try to retain a copy of the GP-MATE patient-held component for the patient’s medical records?**

Prompts: ease/difficulty of doing so, perceived value, acceptability to patients

1. Some questions on preparation for GP-MATE

**Did you take part in the GP-MATE training offered at the start of the project?**

if yes, prompt for experiences of that training: length, content, which roles should be included

**Did you use the GP-MATE staff toolkit?**

If yes, prompt for experiences of content: appropriateness, staff roles, format

**Were you involved with changing any systems at your practice in order to accommodate GP-MATE?**

Prompts: what changed? ease/difficulty, any revisions to roll out plan agreed at SIV? (if present at SIV)

**Did the local hospital changes to discharge summaries in summer 2024 impact on post-discharge care or on GP-MATE?**

Prompts if yes: timeliness, information quality, impact on GP-MATE consults

1. Finally, we’d like your thoughts on the future of GP-MATE

**What is the added value of GP-MATE a) to yourself b) to the practice?**

Prompts (don’t fish for positives): continuity; resource from research to fund the consults; holistic care

**What is the opportunity cost** **of GP-MATE a) to yourself b) to the practice?**

Prompts (don’t overdo the negative): ongoing funding outside research; time pressures; staff shortage

**Is there anything you would change about GP-MATE or the GP-MATE process to make it better for a) yourself or b) your practice?**

Thank you for speaking to me and making time to help our study.
