# Supplementary material for: Pilot of a novel patient-led intervention for postdischarge from hospital management of older patients’ care in general practice
Source: Fam Med Community Health. 2026 Jul 8;14(3):e003981. doi: 10.1136/fmch-2026-003981 (PMC13347910; doi:10.1136/fmch-2026-003981)
Supplement: online supplemental appendix 4 [file fmch-14-3-s004.docx]

GP-MATE Interview Patient/Carer Topic Guide

**Project introduction**

This study is developing a communication tool for older patients who have been discharged from hospital to better enable them to interact with their general practice. We have called our communication tool GP-MATE. We would like your help to get the design of GP-MATE right. As part of the study your general practice has been using GP-MATE. We are interested in how you used it and what you thought about it.

Does that sound clear to you?

Do you have any questions at this stage?

(TAKE WRITTEN CONSENT)

1. Now we’d like to talk about the set-up of your GP-MATE appointment…..

**Tell us about how and when you received your copy of GP-MATE?**

**What was you understanding of how GP-MATE was meant to be used when your received it?**

**Did you fill any information on your GP-MATE prior to speaking to your practice?**

Prompts if negative answer: do you have any difficulties with writing/language barrier/vision that got in the way of using it? Were there other reasons why you didn’t write on it?

Prompts if positive answer: what kind of things did you record on it? Was the process useful to you?

**Were you offered a GP-MATE appointment with your practice?**

If yes,

**Did you have a GP-MATE appointment with staff from your practice?**

If no prompts: did you decline? If so why? (time pressure, other responsibilities, not being able to get hold of the practice)

If yes,

**What kind of appointment was it?**

Prompts: face to face, telephone, video

**How did the appointment format work for you?**

Prompts: was it the format you would have chosen? (if not, why not)

1. Now we’d like to talk about the GP-MATE appointment itself….

**Can you tell me about your experience of using GP-MATE in appointment you had?**

Prompts: did you find it useful? Barriers/facilitators, how did you feel about the outcome from the appointment? changed their relationship with their general practice?

**Did the GP-MATE process change your understanding of anything that happened to you while or after you were in hospital?**

Prompts: medications, diagnoses, follow-up care, did anything change in your care as a result of using GP-MATE?, was it different to previous discharge experiences?

**Did you write any information on your GP-MATE during or after your appointment?**

Prompts: what kind of information did you find useful to record? Was there enough space to write on it? Were there any extra headings you would have liked to see?

**Did writing on your GP-MATE aid your understanding or help you remember things?**

Prompts: whether the process changed their health literacy levels

1. Finally, we’d like your thoughts on the future of GP-MATE

**Would you use GP-MATE again or recommend it to friends/relatives?**

Why? Barriers/facilitators to using it

**Is there anything you would change about GP-MATE or the GP-MATE process to make it better for you?**

**If positive indication at consent - Would you be willing to share your copy of your GP-MATE with the research team?**

*assurance of redacting of any identifiable details, if Yes, proceed to photograph document and redact live with patient/carer present*

CHECK DEMOGRAPHICS INFORMATION COMPLETE before end if not captured by email.

**Your age and ethnicity?**

**Approximately how long you have been a patient at your practice?**

**How often do you tend to have an appointment at your practice?**

**Would you consider yourself to have one or more long term conditions?**

Thank you for speaking to me and giving up your time to help our study.
