# Supplementary material for: Pilot of a novel patient-led intervention for postdischarge from hospital management of older patients’ care in general practice
Source: Fam Med Community Health. 2026 Jul 8;14(3):e003981. doi: 10.1136/fmch-2026-003981 (PMC13347910; doi:10.1136/fmch-2026-003981)
Supplement: online supplemental appendix 5 [file fmch-14-3-s005.docx]

GP-MATE Mini-interview with administrative staff

**Introduction**

As part of the study you have been involved with coordinating GP-MATE appointment booking. We are interested in how that process is going and want to speak to you for about 15 minutes. We might like to speak to you again in 3 months’ time about this depending on how things are going with getting patients into the study.

Does that sound clear to you?

Do you have any questions at this stage?

(TAKE WRITTEN CONSENT)

**How is the rolling search going?**

(prompts: how many patients so far, problems with identifying codes, support from CRN nurses)

**How do you find the call to patients/carers to book the appointment?**

(prompts: does it take long to explain GP-MATE, are people accepting of the idea)

**How is booking going?**

(prompts: has there been sufficient appointment book space created, are other staff involved, are any problems reported back to you)

**How is getting copies of GP-MATE forms back from patients going?**

(prompts: return rate, capturing people on the way out, scanning on)

CHECK DEMOGRAPHICS INFORMATION COMPLETE before end if not captured by email

**How long have you worked at your practice?**

**How many years’ have you been qualified in your current role?**

**Age and ethnicity**

Thank you for speaking to me and making time to help our study.
