# Supplementary material for: Pilot of a novel patient-led intervention for postdischarge from hospital management of older patients’ care in general practice
Source: Fam Med Community Health. 2026 Jul 8;14(3):e003981. doi: 10.1136/fmch-2026-003981 (PMC13347910; doi:10.1136/fmch-2026-003981)
Supplement: online supplemental appendix 6 [file fmch-14-3-s006.docx]

| **Item descriptor** | **Number at least part complete** | **Percentage at least part complete** | **Number indicating that this item is not applicable for them** | **Percentage indicating that this item is not applicable for them** |
| --- | --- | --- | --- | --- |
| Demographics and set up | | | | |
| Patient/carer status | 13/17 | 76.5% | - | - |
| Identifiers | 17/17 | 100% | - | - |
| Carer contact | 14/17 | 82.4% | 6 | 35.3% |
| Relation of carer | 14/17 | 82.4% | 6 | 35.3% |
| Lasting Power of Attorney | 15/17 | 88.2% | 14 | 82.4% |
| Reason for admission | 17/17 | 100% | - | - |
| Expectations of GP-MATE | 13/17 | 76.5% | - | - |
| Continuity | | | | |
| Continuity | 17/17 | 100% | - | - |
| Carers and Caring | | | | |
| Care need | 16/17 | 94.1% | - | - |
| How carer helps | 14/17 | 82.4% | 1 | 5.9% |
| Medical home care | 16/17 | 94.1% | 12 | 70.6% |
| Missing care | 14/17 | 82.4% | 13 | 76.5% |
| Live alone status* | 17/17 | 100% | - | - |
| Loneliness | 17/17 | 100% | - | - |
| Medications | | | | |
| Medicines table | 13/17 | 76.5% | - | - |
| Medicines concerns | 15/17 | 88.2% | 8 | 47.1% |
| Information Power | | | | |
| Ownership of discharge summary** | 16/17 | 94.1% | - | - |
| Talking point on discharge summary | 14/17 | 82.4% | 10 | 58.8% |
| Missing from discharge summary | 14/17 | 82.4% | 10 | 58.8% |
| Questions on coming home | 13/17 | 76.5% | 8 | 47.1% |
| Concerns on coming home | 16/17 | 94.1% | 6 | 35.3% |
| Priorities | 16/17 | 94.1% | - | - |
| Red flags | 12/17 | 70.6% | - | - |

**Appendix 6 – Patient-held GP-MATE completeness**

**Only 5/17 (29%) of the participants actually lived alone ** 14/17 (87.50%) of participants actually had a copy of the discharge summary. Sub-divided sections of the table correspond to the component parts of GP-MATE patient-held tool (22). In columns 4 and 5 a dash (-) indicates this is not appropriate for this question.*
