# Supplementary material for: Pilot of a novel patient-led intervention for postdischarge from hospital management of older patients’ care in general practice
Source: Fam Med Community Health. 2026 Jul 8;14(3):e003981. doi: 10.1136/fmch-2026-003981 (PMC13347910; doi:10.1136/fmch-2026-003981)
Supplement: online supplemental appendix 7 [file fmch-14-3-s007.docx]

General
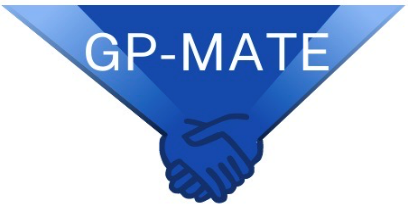
Practice Staff GP-MATE Toolkit

A resource for general practice staff to help improve safety of ongoing care for older patients who are discharged from hospital

Authors:

Dr R A Spencer - *Associate Professor of General Practice,* *PhD, MRCGP*

Dr Z Shariff – *Research Fellow, PhD, MPharm*


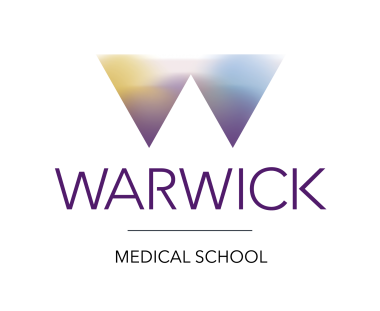


Contents

[Welcome to the GP-MATE staff toolkit 3](#_Toc161830733)

[An Introduction to GP-MATE 4](#_Toc161830734)

[How the toolkit was created 5](#_Toc161830735)

[Getting going with GP-MATE 6](#_Toc161830736)

[What did we learn from current practice for discharge summary handling? 6](#_Toc161830737)

[How will GP-MATE be initiated? 6](#_Toc161830738)

[An admin staff member’s guide to booking a GP-MATE appointment 7](#_Toc161830739)

[An administrative staff member’s guide to the GP-MATE mail-out 8](#_Toc161830740)

[Administrative Team FAQs 9](#_Toc161830741)

[Setting up GP-MATE appointments – a guide for senior management staff 10](#_Toc161830742)

[Which staff role? 10](#_Toc161830743)

[What type of appointment format? 10](#_Toc161830744)

[Continuity of care after discharge 11](#_Toc161830745)

[How GP-MATE might affect electronic document (*Docman*) processing 12](#_Toc161830746)

[The role of administrative team members in Docman processing 12](#_Toc161830747)

[How to document GP-MATE consultations 13](#_Toc161830748)

[Conducting a GP-MATE appointment – A guide for clinicians 14](#_Toc161830749)

[Appendices 21](#_Toc161830750)

[Appendix 1 - Process maps of study practices 22](#_Toc161830751)

[Appendix 2- Teach-back technique 25](#_Toc161830752)

[Appendix 3 - D-CEGRM 26](#_Toc161830753)

[Appendix 4 - Adult literacy problems 27](#_Toc161830754)

[Appendix 5 RCGP continuity toolkit 28](#_Toc161830755)

[Acknowledgements 29](#_Toc161830756)

[Initial Transitional Care Contact - AAFP 29](#_Toc161830757)

[Project RED 29](#_Toc161830758)

[Scottish Patient Safety Programme 29](#_Toc161830759)

# Welcome to the GP-MATE staff toolkit

This toolkit for general practice staff is part of the GP-MATE study*. It is filled with resources to help your practice staff provide safer care for older patients after discharge from hospital. You can use as many (or as few) of the resources as you want to during the pilot phase of testing GP-MATE at your practice. The resources are arranged according to our study priority framework identified by our co-production team of patients, carers, general practice staff and researchers. They will guide you through setting up and conducting GP-MATE consultations.

The toolkit contains other items found in published literature** about safer post-discharge care and from the websites of respected organisations that have accredited functions in making health and social care safer and better.

We would like to hear your feedback on the toolkit and welcome any comments via the survey links in each section.

Thank you for taking the time to look through the resources in this toolkit. We hope they are of use to you during the research project and beyond.

Dr Rachel Spencer

*Academic GP (University of Warwick)*

Dr Zakia Shariff

*Academic Pharmacist (University of Warwick)*

** This toolkit is part of the GP-MATE research study and is under development as part of our package of GP-MATE resources. It remains the intellectual property of the University of Warwick. Copyright © November 2023 Rachel Spencer.* Permission granted to reproduce for personal and educational use only. Commercial copying, hiring, lending is prohibited.

***For a full description of our search strategies please see our published literature review paper and note that this search was updated in winter 2022/23* [*https://www.sciencedirect.com/science/article/abs/pii/S0738399120306741*](https://www.sciencedirect.com/science/article/abs/pii/S0738399120306741)


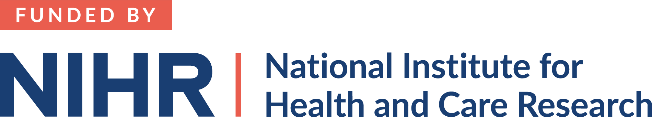

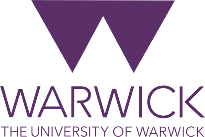


This study/project is funded by the NIHR Advanced Fellowship 301328. The views expressed are those of the author(s) and not necessarily those of the NIHR or the Department of Health and Social Care.

# An Introduction to GP-MATE

**G**eneral **P**ractice **M**anagement **A**fter **T**ransition **E**vents (**GP-MATE**) - Developing an intervention to assist older patients’ communication with their GP practice after discharge from hospital in order to improve patient safety

**Aim:** To make and test an approach (‘GP-MATE’) to assist older patients and their carers to discuss with their GP practice their care after coming home from hospital. This is to make care safer and prevent mistakes from occurring.

**Background**: Being discharged from hospital carries risks. Older people who often have complex ongoing health needs are most at risk, and with over 5 million patients aged 75 or older coming home from English hospitals each year this is an increasingly serious issue. For each patient a letter is sent from the hospital to their GP. This should explain the care that has been provided, and what needs to be done in the weeks and months ahead. There are problems with how many of these letters are managed, and one in thirteen older patients are harmed as a result (over 400,000 patients in England each year). Harms are usually mild, but sometimes include; having to go back into hospital, organ damage and even early death.

Older patients and their carers have a key role to play in preventing these errors and harms. GP-MATE will be designed to help them take a more active role in their general practice care after coming home from hospital. It will help empower them to do so with greater success.

**Design and Methods used:** This 4-year study uses a method called ‘Experienced Based Co Design’, so allowing patients and carers to be closely involved in all stages of developing GP-MATE.

- Year 1 (2022) - The first step is to carefully create a film of patients’ experiences following hospital discharge that depicts the strengths and weaknesses of current practice.
- Year 2 (2023) - To design GP-MATE, three groups of older patients and GP staff across the country will meet. The film will be used to break down barriers within the groups that can prevent working together effectively. We will provide examples of approaches that have been used in other countries to help patients discuss their care with healthcare staff, and work with the groups to create GP-MATE.
- Year 3 (2024) - We will study the use of GP-MATE at eight general practices. We will talk to 24 patients and their carers and 24 staff to gather their views. Everyone who uses GP-MATE will be sent a survey, and we will look at the records of around 300 patients for evidence of its impact.
- Year 4 (2025)- We will draw on our findings to perfect GP-MATE’s design to make it ready for national use and future research aimed at showing how it leads to better outcomes for patients, health services and society as a whole.

**Patient and Public Involvement:** This study is led by patients and carers who will be involved at all stages of the study through a PPI-E panel. This will help to ensure GP-MATE is both useful to and useable by, patients and carers. Two patient events have explored the importance of this topic and informed the research design.

# How the toolkit was created

The first two years of the GP-MATE study are now complete. This staff toolkit is one output of our national co-production process, the other output is our patient-held GP-MATE tool.

The co-production involved 19 older patients/their carers and 17 general practice staff in three groups from across England. We included clinical and administrative staff in our co-production. Our co-production process was triggered by a patient film we made – if you are interested you can watch the film here:

<https://www.youtube.com/watch?v=9x1OgS627yY>

Our co-production team identified 6 main priorities for our work and our patient-held tool and this toolkit are built around them. They are:


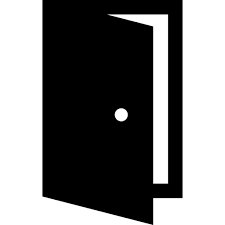
**Overcoming barriers in access to general practice after discharge**


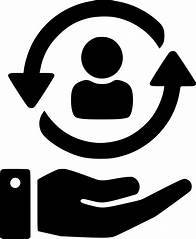
 **Continuity of care after discharge**


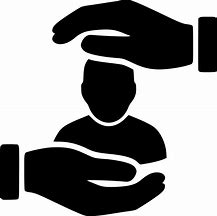
 **Carers and caring after discharge**


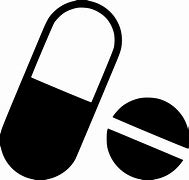
 **Medications after discharge**

**
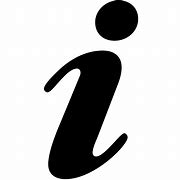
Information power after discharge**

Our film patients were recruited from 10 practices in the West Midlands who allowed their post-discharge care systems to be analysed for the GP-MATE study. We include some process maps of their systems in appendix 1 if you are interested to compare your systems to theirs. Recommendations for best practices based on these systems are included throughout the toolkit.

# Getting going with GP-MATE

##


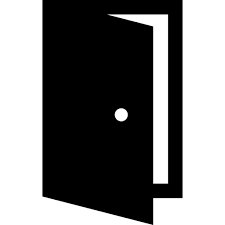
**Overcoming barriers in access to general practice after discharge**

## What did we learn from current practice for discharge summary handling?

What we were also able to determine is that there is currently no standard of care which currently encourages practices to routinely review patients after discharge from hospital (though this has been part of DES incentives in the past). Practices who took part in the first phase of the study are not routinely calling or seeing all older patients post discharge and instead contact is initiated by either the patient (if they have a problem) or by the practice staff (if an action is outstanding). Patients in our trigger film and co-production determined that an over-arching priority for GP-MATE should be to break down barriers in access to general practice for vulnerable older patients after discharge from hospital.

## How will GP-MATE be initiated?

GP-MATE will be initiated when an admin staff member identifies an eligible patient who has been discharged from hospital on the weekly search. Eligible patients are those that are aged 65 or over and who had been admitted to hospital. It is difficult to predict the number of consultations as this will very much depend on your population size and age.

You should aim to book GP-MATE appointments within the coming week. We think GP-MATE will have the potential to have the greatest impact on health outcomes if it is conducted as soon as possible after discharge from hospital. We recognise that this is challenging and here we include some information on how to plan appointment space for GP-MATE.

- How to pick your staff who will offer GP-MATE
- GP-MATE training
- Colour coding GP-MATE appointments to allow administrative staff to recognise them

Once an eligible patient has been identified, the guide in the box below can be used to book a GP-MATE appointment.

## An admin staff member’s guide to booking a GP-MATE appointment

GP-MATE is a new tool to help older patients and their carers out when they come home from hospital. We are testing this tool in our practice. We need you to book patients and/or their carers in for a special appointment and mail out a study pack. Please use a special GP-MATE 20 minute slot with the following practitioners: …………………………………………………………………………………………………………………………………………………………………………………………………………………………………………………………………………………………………………………………..(practice to choose relevant HCP groups that they would like to conduct appointments).

If you want to, you can use a little script we have prepared to explain the appointments to patients/carers. Please look at the banner on the notes to see if the patient has a carer listed as the main point of contact, if so, please address the script below to the carer instead.

“Hello, [patient/carer name]. I’m calling from [surgery name]. We are trying out a new idea of calling all patients aged 65 and over after they’ve come home from hospital. I wondered if you’d like to take up a special appointment that we are offering with one of our [staff role(s)] to talk about your recent hospital stay?

[Pause to allow indication of understanding/early consent or dissent]

“This is part of a research study we are running called GP-MATE. We will be sending an information pack through the post about it. In the pack is a special patient-held document we’d like you to have a go at filling out before the appointment. Can you please also have it with you at the appointment?”

Can I book the appointment for your now?”

[Pause to allow consent/dissent]

**If the patient/carer says that they have an urgent need for an appointment and cannot wait until the GP-MATE slot, please revert to your practices’ usual booking arrangements for this type of need.** Otherwise proceed to book the appointment.

Please ask the patient to write down **who** the appointment is with and the **date/time** (or assure them a text message will be sent with this information if that is your standard practice).

*If it becomes apparent in the call that the patient has new confusion, or a family member not previously listed as a carer is handling calls for them see the FAQ training resources below.*

Please feel free to leave feedback on this guide [here](https://warwick.co1.qualtrics.com/jfe/form/SV_bKqBtE29g7J5dEG)

## An administrative staff member’s guide to the GP-MATE mail-out

1. Pick any GP-MATE pack
2. Open the GP-MATE practice specific excel spreadsheet found here ………………………………………………………………………………………………….[practice to write file path]
3. Find the unique ID number on the header of the paperwork inside the pack. It is a 4-digit number and appears in a box like this:

|  |  |  |  |
| --- | --- | --- | --- |

1. Write the patient’s EMIS number and the unique ID number into one line of the excel spreadsheet like this:

| EMIS number | Unique ID number |
| --- | --- |
| e.g. 12345 | e.g. 1001 |

1. Print address details for the patient and insert into pack with address showing in window.
2. Seal and post out the pack to the patient.

Please feel free to leave feedback on this guide [here](https://warwick.co1.qualtrics.com/jfe/form/SV_2tsY3cvLfIs8qns)

## Administrative Team FAQs

What to do if……..

I’ve called the patient to book the appointment but they don’t answer?

- Try other numbers on the record
- Move on to another patient
- If you cannot recruit to fill the appointments, try to call again

A patient or previously registered carer calls up saying they have received a GP-MATE pack and have missed their appointment call?

- Book the GP-MATE appointment
- DO NOT send out another pack

I’ve called the patient, but I’m worried they seem confused or are not understanding what I’m saying?

- Look for carers’ details on the record to call and invite this person to attend the GP-MATE appointment with their relative if possible.
- Book the GP-MATE appointment with the carer attending alongside the patient unless to do so would cause distress or is impossible
- Post out the GP-MATE pack and tell the carer to expect it at the patient’s address as long as the above is fulfilled

The patient is telling you they would like you to speak to someone else on their behalf, but no carer details are recorded in the notes?

- Book the GP-MATE appointment
- Revert to your policy for registering carers as proxy contacts
- Let the patient know that their new carer is welcome at their GP-MATE appointment
- Post out the GP-MATE pack anyway on this day

If a relative/carer answers the phone who has not previously been recorded in the record as a proxy contact for the patient?

- Ask to speak to the patient directly, if this is not possible….
- Revert to your policy for registering carers as proxy contacts
- DO NOT book the GP-MATE appointment at this time

Please feel free to leave feedback on these FAQs [here](https://warwick.co1.qualtrics.com/jfe/form/SV_5vDFkIsuctWPDGC)

## Setting up GP-MATE appointments – a guide for senior management staff

## Which staff role?

At this stage of the GP-MATE study we are allowing practices to choose which professional role they would like to try out for delivering GP-MATE appointments. In the table below are some pros and cons of different staff roles. Your choice as a practice will depend on the staff resource you have and the level of training of specific individuals in those roles.

| Professional Role | Advantages | Disadvantages |
| --- | --- | --- |
| Pharmacist | Medicines reconciliation specialist | May not have expertise to cope with *information power* section |
| GP/GP trainee | Expertise to cope with all sections of GP-MATE | Opportunity cost |
| Nurse | Career development opportunity for the right nurse with prescribing training | May not have expertise to cope with *information power* section |
| Physician’s Assistant | Career development opportunity for the right PA with prescribing training | May not have expertise to cope with *information power* section |
| Social prescriber | Not recommended for this role* | |
| Occupational therapist | Not recommended for this role* | |

**GP-MATE contains a medications section that we don’t think is appropriate for non-prescribing clinicians to navigate.*

## What type of appointment format?

Older patients who have come home from hospital are often medically and socially vulnerable in the immediate post-discharge period. Some are already classed as medically frail or are housebound. In order to best serve this population it is likely that different formats of GP-MATE appointment will be necessary. If the patient is able to attend the practice there are clear benefits to conducting GP-MATE as a face-to-face appointment. If the patient cannot attend the practice there are two options:

1. Book the patient a home visit GP-MATE appointment
2. Book the patient a GP-MATE telephone call appointment

Fit this choice around your current home visit booking systems. You can instruct your administrative teams to book them a home visit for the GP-MATE appointment if you think this would be beneficial for the patient. This might require your administrator who is running the search to discuss with a clinician about whether a visit is indicated.

Please feel free to leave feedback on this section [here](https://warwick.co1.qualtrics.com/jfe/form/SV_aWbyJiliaq1uQWq)

**
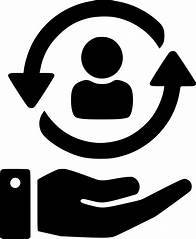
 Continuity of care after discharge**

## Continuity of care after discharge

Continuity of care after discharge was a priority for patients in our co-production process. We therefore encourage patients taking part in GP-MATE to record the name of the clinician who their GP-MATE appointment was with. As you will see in the ‘An admin staff member’s guide to booking a GP-MATE appointment’ tool above we want your reception staff to facilitate patients to record this information. Additional resources can be found in the RCGP continuity toolkit (Appendix 5).

In our study of West-Midlands practices, we found a range of techniques were employed for sending discharge summaries through to a clinician, see table below. Some of these focus on continuity and others prioritise other elements (speed, ‘fairness’ and expertise). We encourage you to think about the current system set-up in your practice and the motivations behind its design.

| **Priorities continuity** | **Prioritises other elements** |
| --- | --- |
| patient’s regular GP, “who the patient is under,” Nominated GP/Named GP | Random clinician selected (speed) |
| Clinician that referred patient to hospital | Central inbox dealt with every day (speed) |
| Clinician that saw the patient most recently | Whoever is in the next day (speed) |
| GP that looks after nursing homes | Sharing out (ensure workload distributed evenly) |
| Use of a buddy system when relevant clinician on holiday | Discharge summaries with medication changes sent to pharmacists (MR expertise) |
|  | GP with interest in health topic area (expertise) |

We have two best practice pointers arising from our work in practice systems:

| **Safety issues practices raised with us** | **Best practice for attending to the issue** |
| --- | --- |
| Getting the discharge summary to the ‘right’ clinician | Administrative staff processing *Docman* should read the record to determine who saw patient last or who is patients’ usual GP |
| **Balanced against……….** | |
| Getting the discharge summary to the clinician in a timely way | Admin staff hold an up-to-date chart with staff leave and normal working days for refence while disseminating *Docman* |

## How GP-MATE might affect electronic document (*Docman*) processing

The discharge summaries for the patients who are part of the GP-MATE study will still be arriving on the electronic download to your practices’ global inbox. Ability of your staff to view the discharge summary is an integral part of the GP-MATE consultation. A common audit target for actioning discharge summaries is 2 working days. Your normal filing systems should be followed while the study is ongoing. We don’t feel it is practicable to try and ask you to divert *Docman* to the clinician who is carrying out the GP-MATE appointment for two reasons:

1. GP-MATE searches may well occur after the discharge summary has already been sent to a clinicians’ inbox
2. We don’t want to change any systems you already have in place for continuity

Instead, we ask that you make your clinical staff who receive *Docman* aware that if a discharged patient is aged 65 or older, they will be offered a GP-MATE appointment. Clinicians may choose to send on the summary to the clinician who is conducting the appointment (if they also receive *Docman*) or they may choose to retain clinical responsibility. This choice, perhaps, will depend on the urgency of the medical information contained in the discharge summary.

## The role of administrative team members in Docman processing

Some practices use their highly trained administrative staff to undertake specific roles in relation to discharge summary processing. Most of these clinical assistants had been trained in buddying models with experienced GPs and had undergone a period of highly supervised working before being trusted to assume responsibility. This work spares clinical staff time and can ensure more accurate/consistent coding of information. It may not be right for your practice; it depends on the staff resource that you have and the level of training you are willing to undertake for selected administrative staff.

| **Safety issues practices raised with us** | **Best practice for attending to the issue** |
| --- | --- |
| Ensuring medication changes are identified and addressed | Admin staff identify discharge summaries with medication changes and send these through to the pharmacist (if available) or GP |
| Ensuring new diagnoses/co-morbidities are recorded | Admin staff undertake Read coding when going through the summary. Any new diagnoses/co-morbidities are highlighted on the discharge summary using the highlighter function within *Docman* so an audit trail can be determined for the ‘problem’ list. Summaries containing significant diagnoses (especially cancer) are sent on to a clinician. |
| Ensuring all actions identified on the discharge summary are followed up | Admin staff highlight actions on the summary using the highlighter function within *Docman*. The summary is sent on to the clinician for them to action if appropriate. |

We have several best practice pointers arising from our work in practice systems:

**
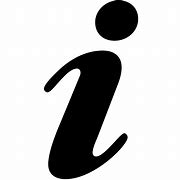
Information power after discharge**

## How to document GP-MATE consultations

This is ultimately up to the clinicians which you decide to use to deploy GP-MATE. It would be possible to construct a documentation template for EMIS if you feel that staff would need or welcome the assistance of a template. We encourage you to photocopy the patient’s own patient-held GP-MATE form as they leave the practice and scan this into their medical record. GPs in our co-production teams felt that this document could be very useful as it contains information not routinely found within the patient’s electronic health record.

# Conducting a GP-MATE appointment – A guide for clinicians

This section of the toolkit follows the order of the questions on the patient-held GP-MATE. It contains useful information for clinicians on:

1. how to guide patients through this section if they need help
2. how to answer patients’ queries arising from this section
3. how to keep the consultation on track

You have a 20-minute appointment for GP-MATE, and this includes any time you want to spend documenting the appointment. You can use the GP-MATE documentation template if your practice has set it up. We have not told patients about the length of the appointment.

The patient-held GP-MATE contains the following sections:

**An introduction**


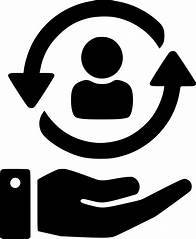
 **Continuity of care after discharge**


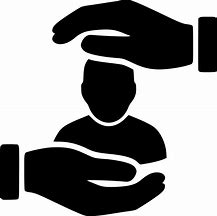
 **Carers and caring after discharge**


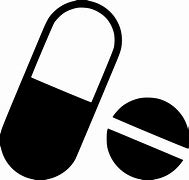
**Medications after discharge**

**
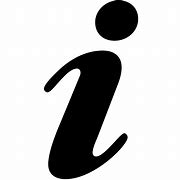
Information power after discharge**

You need to attend to all sections of the patient-held tool during the GP-MATE appointment but if the patient has multiple problems or complex needs it is OK to book them another ordinary appointment as you see fit.

**An introduction**

**Please feel free to leave feedback on this section** [**here**](https://warwick.co1.qualtrics.com/jfe/form/SV_b9rYff0Iy0RNPhA)

The first page of the patient-held GP-MATE tool contains carer details – look carefully at the banner of the electronic health record and if these carer details are new:

1. Ask the patient if they’d like these details to be transferred to their medical record? Ask carers whether they are capable and comfortable in providing care. Check if they are a new carer who is starting up care after discharge. Signpost to further resources (provided on GP-MATE patient-held tool). Ask if they have any further concerns or questions as a carer
2. Facilitate reception staff to capture a copy of the patient-held GP-MATE at the end of the appointment in order to retain these details. Send the patient to the desk at the end of the appointment so they can take a copy for the medical notes.
3. Don’t feel the need to transfer the details to the electronic record yourself – you probably won’t have time to do this in the 20 minute consultation

The first page also contains details of Lasting Power of Attorney.

1. Many patients don’t understand what LPA is, have a basic understanding yourself of the difference between LPA for health and welfare and LPA for finances.
2. The patient-held tool points patients to the following link, point this out and consider having a look yourself if you are unsure: <https://www.nhs.uk/conditions/social-care-and-support-guide/making-decisions-for-someone-else/giving-someone-power-of-attorney/>
3. Offer to record the LPA decision but don’t offer to sign LPAs. Point patients instead to the Office of the Public Guardian <https://www.gov.uk/government/organisations/office-of-the-public-guardian>

Reason for admission to hospital:

1. Check this matches the reason given in the discharge summary
2. If it doesn’t match, then tell them the reason given in the discharge summary in plain English and explore any confusion you find
3. You can only tell them what you can glean from the discharge summary – explain the limitations of communication from the hospital and move on

What do you hope to get out of your GP-MATE consultation?

1. We anticipate that some patients won’t write anything here, despite the instruction at the end to come back to this question after exploring all domains on the patient-held tool
2. If there is nothing written in this section move into the rest of the tool. If something is written in this section, assure the patient that you will hold this information in mind as you move through the appointment
3. Don’t try to answer questions at this point – move through the tool in order but keeping the central patient priority in mind (hopefully this question will save time overall – you might recognise it as ICE – Ideas, Concerns and Expectations)

**Please remember you can leave feedback on this section** [**here**](https://warwick.co1.qualtrics.com/jfe/form/SV_b9rYff0Iy0RNPhA)


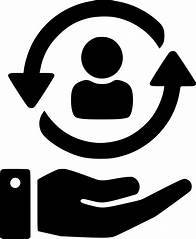
 **Continuity of care after discharge**

**Please feel free to leave feedback on this section** [**here**](https://warwick.co1.qualtrics.com/jfe/form/SV_bw5A0d4UK4c0UQu)

My GP-MATE appointment is on:……………………(date) at ………………(time) with: ………………….

1. This section is designed so the patient can refer back to a written record of your name. Help the patient if they have spelt it incorrectly.
2. You might like to help the patient put in today’s date if they have not done so, tell them that’s so they have a record of when this discussion took place so that they can refer back to it if they want to
3. Recording of the time provides the patient with a written record of when the consultation took place.

Days they (you) usually work:

1. We recognise that sharing this information with patients may be a new concept – rest assured that patients have been given written information that they cannot always expect to get hold of you on these days and also that sometimes urgency is more important than continuity.
2. Share your usual working days if you feel able and want to do so.
3. Don’t reiterate safety caveats verbally – patients understand the limitations of continuity better than you think they do.


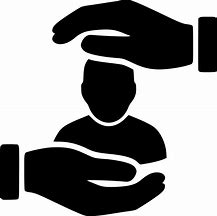
 **Carers and caring after discharge**

**Please feel free to leave feedback on this section** [**here**](https://warwick.co1.qualtrics.com/jfe/form/SV_br9Q3rJHltACOGO)

Is anyone helping you out since you came home from hospital?

1. A “ yes” answer here prompts the patient for more detail
2. If the patient has ringed “No” and you think they clearly need some help, explore more in this section.
3. If the patient has ringed “No” and you feel that’s appropriate for them, move on to question 2. on loneliness

Who is/are the key person/people who help you out and how do they help you out?

1. Look to see if this information is already contained in the medical record, if not, think how you want to document it for ease of access/future reference.
2. If it is particularly important for the patient, you might create a support map to illustrate the support a patient has, e.g. D-CEGRM (see appendix 3)
3. You don’t need to identify and capture every source of support – get a key person and then move on

Are you receiving any medical care at home? (E.g., wound care, district nurse visits)

1. Discharge summaries rarely contain this information, but it can be critical to understanding reasons why tenuous discharges may fail and result in readmission.
2. Look for obvious holes in the package of medical care e.g. unable to self-administer insulin and arrange help (if it is required) by onward referral to services
3. Don’t fish for medical care needs where clinically you do not feel there should be any, if this section is blank move on. Feel able to refute a medical care need if you think the request from the patient is unreasonable or out of the range of what the NHS can normally provide.

Is there anything that you feel is missing from the care that you are currently receiving?

1. Discharge summaries rarely contain this information and patients seldom volunteer it unless directly asked. It’s rare that we ask patients to volunteer this information and there’s a great deal of trust involved in what we do with the answer once we’ve obtained it.
2. Look for obvious holes in the package of social care and think about how to encourage patients/carers to contact social services themselves. Point out the links in the patient-held GP-MATE <https://www.carersuk.org/> <https://www.ageuk.org.uk/> <https://store.redcross.org.uk/pages/categories> (independent living equipment). In special circumstances you might want to make a referral yourself to social services or to your social prescribing/occupational therapy services if you have that resource.
3. If this section is blank move on. Feel able to refute a social care need if you think the request from the patient is unreasonable or out of the range of what the council can normally provide.

Do you live alone?

1. A screening question.
2. Being alone is not the same thing as being lonely. When a patient is living alone after a complex discharge this may trigger medical concerns for you as a practitioner.
3. Discuss how the patient is coping if, and only if, you are concerned about them being alone.

Do you want to talk about loneliness?

1. We don’t usually ask patients this question in a medical arena, but it was an important theme from our co-production.
2. Be prepared to point out the resources in the patient-held tool  [Age UK](https://www.ageuk.org.uk/information-advice/health-wellbeing/loneliness/) [The Silver Line Helpline](https://www.thesilverline.org.uk/) <https://www.befriending.co.uk/directory/> <https://www.reengage.org.uk> and add any sources of support that you have locally. Consider social prescribing referral if you have that resource.
3. If a patient has ringed “no” then respect that choice and don’t feel the need to enquire further. If mental health problems are uncovered give yourself licence to book another appointment with the patient – it is not possible to sort everything out in the GP-MATE appointment.

**Please remember you can leave feedback on this section** [**here**](https://warwick.co1.qualtrics.com/jfe/form/SV_br9Q3rJHltACOGO)


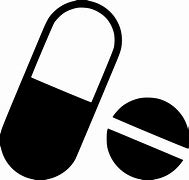
 **Medications after discharge**

**Please feel free to leave feedback on this section** [**here**](https://warwick.co1.qualtrics.com/jfe/form/SV_6s7l5ZYjb0ssjoq)

There are lots of medication reconciliation tools in literature and online. We’re going to concentrate on the GP-MATE patient-held version first so you know what to expect from your patient in the appointment. In the acknowledgments section, we have listed some examples of other medication reconciliation tools that you could use in your practice. We are only interested in here in REPEAT medicines that are NEW, have CHANGED DOSE or have STOPPED.

The patient-held GP—MATE medications table looks like this:

| Name of medication | Newly started in hospital (tick) | Changed dose (tick) | Stopped  (tick) | What is/was it for? | Why was it added/changed/stopped? | Temporary (T) or Permanent (P) change? |
| --- | --- | --- | --- | --- | --- | --- |
|  |  |  |  |  |  |  |

1. They may have completed none, some, or all of the required changes listed on the discharge summary. Pay particular attention to where what the patient has recorded is different to what the discharge summary says – try to determine which version is truth. Also pay attention to incomplete boxes – ask your patient why they didn’t complete it – do they not know the answer?
2. Supply them with the correct answers and then use teach back method (see appendix 2) or another method you are comfortable with to check they’ve understood.
3. You have to choose with your patient whether you’re going to write, or they are and whether this will take place during or after the consultation. Consider that patients take more time to write and you may have to spell drug names for them.

Is there anything that you are concerned about in relation to your medication since coming home?

1. N/A
2. You might be able to answer their queries directly or you might direct them to a practice or community pharmacist. You might need to discuss with your colleagues, especially if the medicine is new or specialist initiated. If their concerns related to a new repeat medicine then draw their attention to the [https://www.england.nhs.uk/primary-care/pharmacy/pharmacy-services/nhs-new-medicine-service/](https://www.england.nhs.uk/primary-care/pharmacy/pharmacy-services/nhs-new-medicine-service/%20) as signposted in the patient-held GP-MATE. Sometimes the SPC leaflet that comes with the medication can be helpful.
3. If there are multiple medicines changes then sometimes the hospital will already have referred the patient to the discharge medicines service <https://www.england.nhs.uk/wp-content/uploads/2021/01/B0366-discharge-medicines-toolkit.pdf>

General practices can’t refer people to this service unfortunately.

**Please remember you can leave feedback on this section** [**here**](https://warwick.co1.qualtrics.com/jfe/form/SV_6s7l5ZYjb0ssjoq)

**
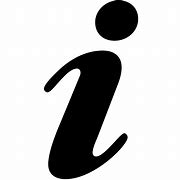
Information power after discharge**

**Please feel free to leave feedback on this section** [**here**](https://warwick.co1.qualtrics.com/jfe/form/SV_6gpKWdCVaFjIm1g)

Information power is the name we have given within the project to the concept that patients are empowered by having access to copies of their health information. This section of the patient-held tool is an opportunity for patients to check their own understanding of the information that has been provided in the discharge summary. Some patients will have many questions relating to this section, some patients will have few or none. It depends on the health literacy and personality of the patient as to how long you will spend on this section of the tool. We recognise that this set of questions may put an onus on you to take on any perceived deficiencies in the care provided by the hospital. We can only do what we feel is within our capabilities in helping the patient to navigate the complexities of NHS systems.

Do you have a copy of your discharge summary letter from your hospital admission? Yes/No

A simple question, but what to do if they answer “No”?

1. You will have the discharge summary open already during the consultation as you’ll have to read it prior to the appointment.
2. Ask if they’d like a copy? Print one out for them (consider the rare circumstances where information in a discharge summary might harm a patient)
3. Tell the patient that this copy is for their own records, do not encourage them to read it now during the appointment.

Is there anything in the discharge letter which you wish to talk about at your GP-MATE appointment?

1. We intended patients to express their lack of understanding in this space but they may use it to speak in a wider sense about the admission.
2. Try to put the emphasis on this space as a health literacy improvement opportunity for the written communication rather than a dissection of what happened in the hospital.
3. It is OK to move briefly through this section, especially when the patient has left it blank.

Do you feel there is anything missing from your discharge letter?

1. N/A
2. Listen for patient safety concerns raised by the patient – especially regarding follow-up tests/investigations that may not have happened as planned.
3. It is OK to move briefly through this section, especially when the patient has left it blank.

Do you have any questions about the follow up healthcare (e.g., tests, follow-up appointments) that have been described in the discharge letter?

1. If the discharge summary lists any test/follow-up or referrals find out if these have happened or if the patient knows when they will happen. Consider the SPSP results tool in the acknowledgements section.
2. Listen for patient safety concerns raised by the patient regarding follow-up tests/investigations that may not have happened as planned.
3. It is OK to move briefly through this section, especially when the patient has left it blank.

Is there anything you are concerned about in relation to either your physical or mental health since coming home?

1. N/A
2. Be led by the patient’s concerns but remember that some problems will need a dedicated second appointment.
3. Use your clinical knowledge to judge the urgency of the presented problems and only deal with what you need to deal with immediately / what you can cope with safely in the GP-MATE appointment.

What is your health priority after coming home from hospital?

1. N/A
2. Tell the patient/carer that you will remember their priority when making decisions about their care.
3. It’s OK if the patient/carer does not have a priority (move on). If the patient has more than one priority try to help them identify the main one for them.

What does your practitioner want you to keep an eye on?

1. You will perhaps be more familiar with this concept as ‘red flags’. We deliberately don’t use that term with patients/carers as it can be worrying.
2. Ask the patient to document in a few simple words the red flag problems that you want them to look out for e.g. fever/swelling. Most patients are not used to writing notes during an appointment so this will be a new concept for them. You need to be alert to adult literacy problems (see appendix 4 for help).
3. Consider writing in this section for the patient if physical impairments/literacy levels or language barrier make it difficult for them to do this themselves in a timely manner. Remember, they have to be able to read what you have written.

**Please remember you can leave feedback on this section** [**here**](https://warwick.co1.qualtrics.com/jfe/form/SV_6gpKWdCVaFjIm1g)

# Appendices

## Appendix 1 - Process maps of study practices

**Practice type A – GP led model**

In this type of model, GPs would receive all discharge summaries and perform the majority of tasks.

| **Positives** | May help to ensure episodic continuity |
| --- | --- |
|  | Potential to tighten patient safety |
|  |  |
| **Negatives** | Increased time pressure on GPs |
|  | Potential for duplication of workload |
|  | May lead to delays in processing discharge summaries |


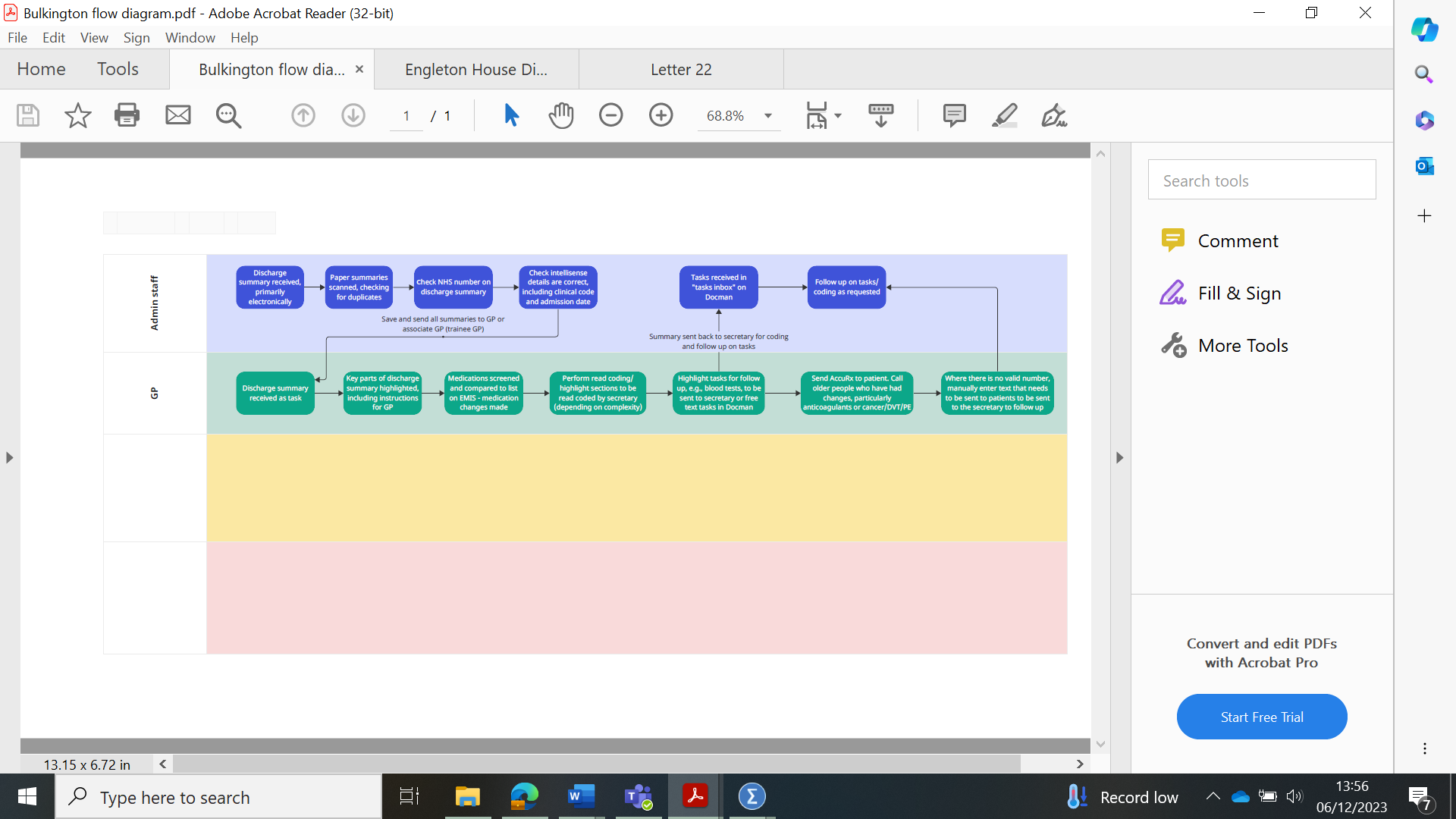
Example:

**Practice type B – Pharmacist led model**

In this type of model, pharmacists (and pharmacy technicians where available) had an important role to play, especially in relation to medication reconciliation.

| **Positives** | Medication reconciliation specialist, therefore potential to tighten patient safety |
| --- | --- |
|  | Reduce pressure on GPs |
|  | Increased potential for contact with patient due to dedicated slots |
|  |  |
| **Negatives** | Not always available at all practices |
|  | Potential for duplication of workload depending on level of trust in pharmacist actions and decisions |
|  |  |

Example:


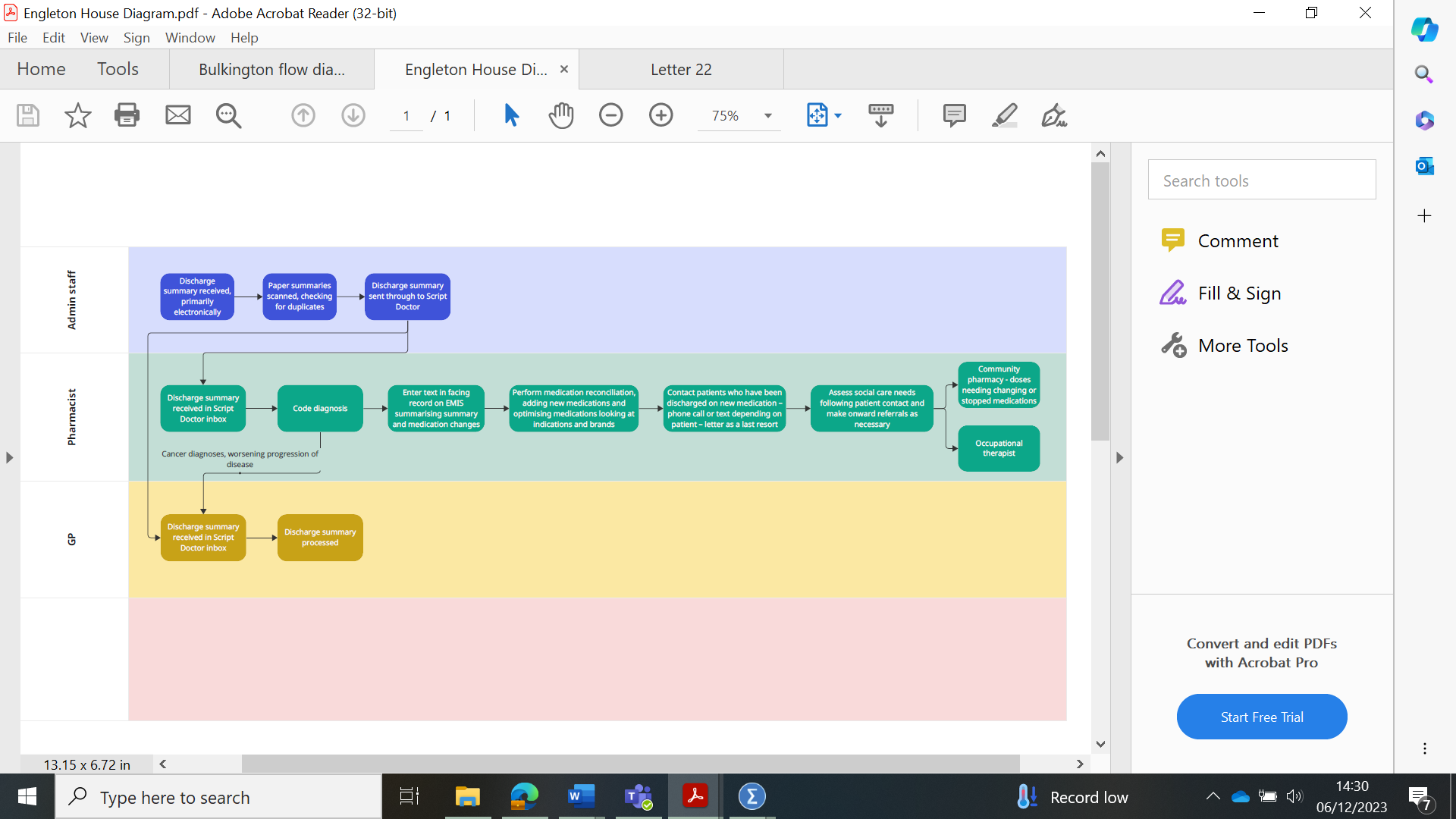


**Practice type C – Administrative led model**

In this type of model, administrative staff take more of a lead role, performing the majority of tasks in relation to discharge summaries and only sending on to a clinician when necessary.

| **Positives** | Reduced pressure on clinicians |
| --- | --- |
|  | Opportunity for enhanced training for administrative staff |
|  |  |
|  |  |
| **Negatives** | Potential for backlog when key administrative staff not in |
|  | May increase pressure on administrative staff when there are highly complex cases |
|  | Increased time pressure on administrative staff balancing a number of roles |


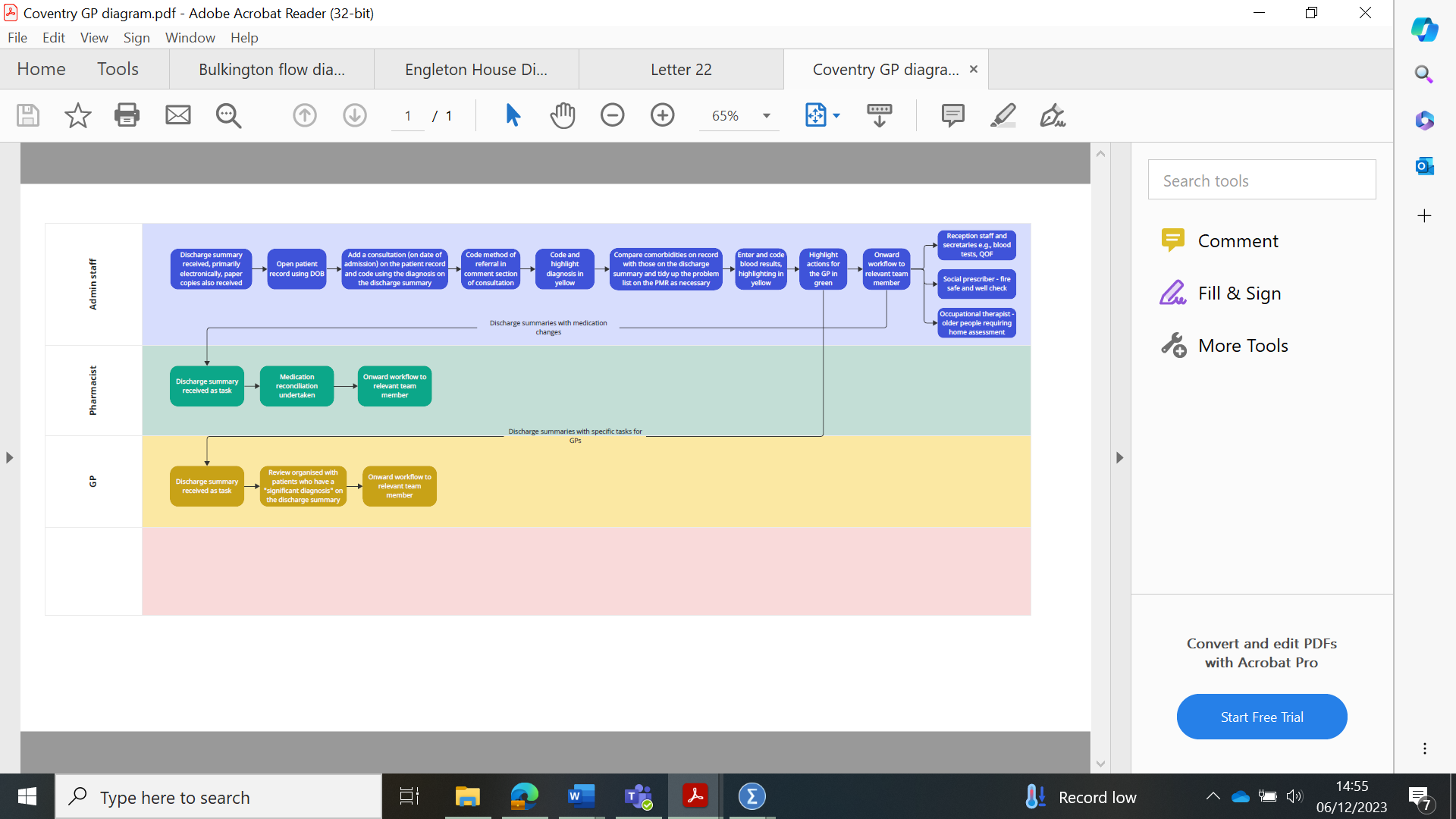
Example:

## Appendix 2- Teach-back technique

**What is the teach-back technique?**

Teach-back is a way to make sure you’ve explained information clearly to a patient or carer. It’s used to check understanding and, if needed, provides you the opportunity to re-explain and check again.

**How to implement teach-back?**

Teach-back involves asking a patient (or carer) to explain in their own words what they need to know or do, in a caring way.

**Trying teach-back with GP-MATE**

- Plan your approach. Think about how you will ask your patients to teach-back. For example, “We have covered a lot of information today, and I want to make sure that I’ve explained everything clearly. Let’s review what we’ve covered so far. Please can you describe the things you need to keep an eye on after your stay in hospital?”
- "Chunk and Check." Instead of waiting until the end of the GP-MATE consultation, try initiating teach-back after each section of the tool.

**Some top tips**

- Try to use a caring tone of voice to make the patient or carer feel comfortable
- Use plain language
- Try to use open-ended questions
- Emphasise that the responsibility is on you, the provider, to explain things clearly
- If the patient or carer isn’t able to teach-back, explain again and re-check
- If possible, use patient-friendly printed materials to support information being provided
- Involve any carers or family members who might be present

References:

- 10 Elements of Competence for Using Teach-back Effectively. Always Use Teach-back. Available at: [Teach Back - Observation Tool Proof 6.indd (higherlogicdownload.s3.amazonaws.com)](http://higherlogicdownload.s3.amazonaws.com/HEALTHLITERACYSOLUTIONS/b33097fb-8e0f-4f8c-b23c-543f80c39ff3/UploadedImages/docs/Teach_Back_-_Observation_Tool.pdf) [Accessed 13^th^ December 2023]
- Health Literacy Universal Precautions Toolkit, 2^nd^ Edition. Use the Teach-Back Method: Tool #5. Available at: [Use the Teach-Back Method: Tool #5 | Agency for Healthcare Research and Quality (ahrq.gov)](https://www.ahrq.gov/health-literacy/improve/precautions/tool5.html) [Accessed 13^th^ December 2023]

## Appendix 3 - D-CEGRM

**What is the D-CEGRM Social Resource Interview?**

- The Coloured Eco-Genetic Relationship Map (CEGRM) is a simple process which helps patients communicate the different types of support they receive e.g.: informational support, emotional support, spiritual/religious support.
- It allows for social support both within and outside families to be identified.
- It has been adapted and tested in a population of patients with complex diabetes, resulting in the D-CEGRM

**
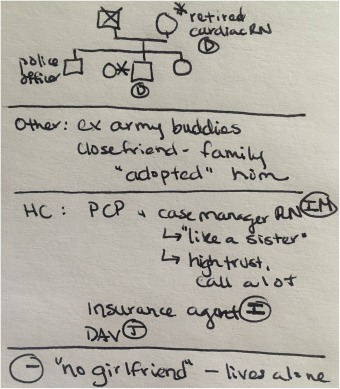
Example D-CEGRM map**

Process to create the D-CEGRM map:

1. Identify family members providing support (anyone particularly helpful is highlighted with a star)
2. Other sources of support identified, e.g., neighbours, co-workers, friends, support groups, church groups
3. Healthcare sources of support identified, e.g., home health nurses
4. People or issues that have a negative influence on health identified

*NB: As this was focused specifically on diabetes management, the questions identifying sources of support were reframed to identify those who helped in four areas related to diabetes self-management: diet, insurance, medication and transportation.*

**Interpreting the map**

Clinical judgement can be used to categorise patients as having inadequate or adequate social support:

| Inadequate support | No primary support person, few sources of additional support, struggling with health-related needs, “negatives” identified (e.g., caretaking duties) |
| --- | --- |
| Adequate support | Has a primary support person, has other sources of support, no report of any relationships that negatively impact self-care, no reports of difficulties navigating healthcare system |

**Adapting D-CEGRM for use in GP-MATE**

- Questions 1, 1a and 1b aim to identify sources of social support on discharge
- If this area is particularly important for the patient, it might be helpful to illustrate these sources of support by creating a support map similar to the D-CEGRM map
- A key output would be to identify whether the patient or carer has adequate or inadequate support, and to make onward referrals as necessary

Reference:

Wallace AS, Pierce NL, Davisson E, Manges K, Tripp-Reimer T. Social resource assessment: Application of a novel communication tool during hospital discharge. Patient Education and Counseling. 2019;102(3):542-9.

## Appendix 4 - Adult literacy problems

**What is literacy?**

“The ability to read, write, speak and listen in a way that lets us communicate effectively and make sense of the world.”^1^

**What is health literacy?**

“The degree to which individuals can obtain, process and understand the basic health information and services needed to make appropriate health decisions.”^2^

**Why is literacy important?**

7.1 million people in the UK can be described as having “very poor literacy skills.” ^1^ This means that reading information from unfamiliar sources or on unfamiliar topics (such as those that may arise during a consultation at general practice) can cause problems.

**The importance of literacy during the GP-MATE consultation**

The patient’s literacy levels may impact their ability to fill out a number of sections in GP-MATE. These include the final question in the introduction on what they hope to get out of GP-MATE, the medication table and the final question in GP-MATE, where the patient is asked to document in a few simple words the red flag problems that have been identified during the consultation. Depending on the individual patient’s literacy levels, this may be difficult for them to do.

**How can we identify a patient’s level of health literacy during a consultation?**

Heuristic indicators to assess a patient's level of health literacy^3^:

- Poor recall of medication name, indication and dosage
- Poor recall of verbal instructions
- Poor recall of written medicine information
- Limited use of medical terms
- Not seeking new information
- Not asking questions

The **teach-back technique** (Appendix 3) is a key tool to determine the patient’s literacy levels.

**How to help patients better understand health information?**

A study^3^ looking at how doctors make themselves understood in primary care consultations found the following techniques were used:

- Use plain language and tools (e.g., images)
- Ask the patient non-shaming open ended questions
- Convey interest through non-verbal communication, e.g., body language, eye contact

*^1^Adult literacy. The National Literacy Trust. Available at:* [*Adult literacy | National Literacy Trust*](https://literacytrust.org.uk/parents-and-families/adult-literacy/) *[Accessed 14^th^ December 2023]*

*^2^Ratzan S, Parker M. Introduction: Health Literacy. 2000. National Library of Medicine. National Institute of Health.*

*^3^Duell P, Clark A, Cooper N, Wright D, Hartt J, Budd T, Bhattacharya D. Final Report for the Determining Patient Health Literacy during a Medicine Consultation Study. Available at:* [*Determining-Patient-Health-Literacy-during-a-Medicine-Consultation-Study-1.pdf (pharmacyresearchuk.org)*](http://pharmacyresearchuk.org/wp-content/uploads/2017/01/Determining-Patient-Health-Literacy-during-a-Medicine-Consultation-Study-1.pdf) *[Accessed 14^th^ December 2023]*

## Appendix 5 RCGP continuity toolkit

The RCGP have put together a continuity of care toolkit for practices looking to improve continuity of care that can be downloaded here:

[Continuity of care: Download the toolkit | RCGP Learning](https://elearning.rcgp.org.uk/mod/book/view.php?id=12895&chapterid=536)

Once downloaded, you can work through the toolkit at a pace suitable for your own practice. The toolkit is divided into three main sections:

- 6 Steps: The guided approach to improving continuity
- Resources: Helpful tools and editable templates to support the steps
- GP practice stories: Examples of what others have done

Different approaches for continuity of care

- **Patients –** You may choose to improve continuity of care for all patients, OR cohorts of patients who would benefit most
- **Workforce –** You may choose to have one GP with overall responsibility for a patient, OR have a micro team where GPs, buddies and a multidisciplinary team look after the patient

Types of continuity of care:

1. **Relational** - Building good patient-professional relationships
2. **Episodic** - GP/team provide continuity of care during an episode of ill health. This is the aim of GP-MATE.
3. **Informational** - Good record keeping to ensure others also understand the patient and their condition
4. **Managerial** – Co-ordinating the patient’s care when a team of multiple professionals are involved

The toolkit identifies the following cohorts of patients that benefit most from continuity initiatives. As you can see, all of these have a strong crossover with the GP-MATE cohort (Page 49 of toolkit)

| **Cohort** | **Considerations** |
| --- | --- |
| Frailty | Patients with Frailty Index >0.36 and a Rockwood Score 7-9 would be in high need of continuity.  Using the Low Frailty Index score may be misleading as a patient may have a high score but be independent and happy with less continuity.  The Cohort of Patients may change - it only takes a single deficit to be severe to render a patient extremely vulnerable and in high need of continuity |
| Polypharmacy | Assess the opportunity to provide continuity of care for this cohort as a micro-team by including the GP and practice pharmacist. |
| Older patients | Age on its own is not a good indicator of continuity of care. Age can be used with another marker e.g. care home, housebound or frailty index |

# Acknowledgements

The following sources were used during the development of GP-MATE. We have summarised the key purpose of each of the tools and provided a link to access for further information.

## Initial Transitional Care Contact - AAFP

This tool was developed by the American Academy of Family Physicians and was used in our co-production meetings to illustrate how the initial contact with patients has previously been recorded.

Available at: [Initial Transitional Care Contact (sonehealthcare.com)](https://sonehealthcare.com/wp-content/uploads/2022/02/TOC-Checklist.pdf)

## Project RED

This project aimed to develop and test a set of activities and materials to improve the discharge process, which they called the Re-Engineered Discharge (RED). The activities are actions that the hospital should undertake before and after a patient’s stay, therefore are not directly related to general practice, but the project was referred to in our co-production meeting to illustrate the various areas post discharge that need to be addressed.

Available at: [Tool 1: Overview | Agency for Healthcare Research and Quality (ahrq.gov)](https://www.ahrq.gov/patient-safety/settings/hospital/red/toolkit/redtool1.html#Purpose)

## Scottish Patient Safety Programme

1. Implementing safe and reliable systems for communication across interfaces of care

A change package was developed to identify interventions that collectively improve the whole system of care. Interventions specifically for primary care in relation to outpatient communication include:

- Ensuring that the letter has been actioned by the appropriate clinician within **2 working days**
- **Clearly implementing** the change in the management plan

**Notifying the patient** of the change in the management plan

Available at: <https://ihub.archive.nhsscotland.net/media/1102/20150109-reporting-template-guidance-with-measurement-plan-v0-2.pdf>

1. Examples of communication terms about test results

Example comments were developed for clinicians to communicate what results mean and whether actions need to be taken or not.

Available at: <https://ihub.archive.nhsscotland.net/media/4767/spsp-pc-data-toolkit-version-may-2015.xlsx>

1. Medication reconciliation

The SPSP has developed a care bundle for medication reconciliation in General Practice, including a list of 5 measures and how to apply these to optimise patient care.

Available at: https://ihub.archive.nhsscotland.net/media/4767/spsp-pc-data-toolkit-version-may-2015.xlsx
